# Supplementary material for: A single-domain green fluorescent protein catenane
Source: Nat Commun. 2023 Jun 13;14:3480. doi: 10.1038/s41467-023-39233-7 (PMC10264447; doi:10.1038/s41467-023-39233-7)
Supplement: Supplementary file 1 — Supplementary Information [file 41467_2023_39233_MOESM1_ESM.pdf]

Supplementary Information

A Single-Domain Green Fluorescent Protein Catenane

Zhiyu Qu[1,2,3,4], *Jing Fang*[1,2,3,4], *Yu-Xiang Wang*[1,2,3,4], *Yibin Sun*[1,2,3,4], *Yajie Liu*[1,2,3,4], *Wen-Hao Wu*[1,2,3,4], *Wen-Bin Zhang*[1,2,3,4,5]*

[1] Beijing National Laboratory for Molecular Sciences, Beijing, P. R. China

[2] Key Laboratory of Polymer Chemistry & Physics of Ministry of Education, Peking University, Beijing, P. R. China

[3] Center for Soft Matter Science and Engineering, Peking University, Beijing, P. R. China

[4] College of Chemistry and Molecular Engineering, Peking University, Beijing, P. R. China

[5] Beijing Academy of Artificial Intelligence, Beijing, P. R. China

Tel.: + 86 10 6276 6876; Fax: + 86 10 6275 1710

E-mail: wenbin@pku.edu.cn

Table of Contents

Sequence Information..2

Supplementary Figures..9

Supplementary Tables..32

Supplementary References..34

Source Data for Supplementary Figures..35

Sequence Information

IntC-GFP1-IntN-GFP2
001 MKGSSIKIATRKYLGKQNVYDIGVERDHNFALKNGFIASNCFNGGENLYFQ/GASMSKGEE
061 LFTGVVPILVELDGDVNGHKFSVRGEGEGDATIGKLTLKFICTTGKLPVPWPTLVTTLTY
121 GVQCFSRYPDHMKRHDFFKSAMPEGYVQERTISFKDDGKYKTRAVVKFEGDTLVNRIELK
181 GTDFKEDGNILGHKLEYNFNSHNVYITADKGTCLSYETEILTVEYGLLPIGKIVEKRIEC
241 TVYSVDNNGNIYTQPVAQWHDRGEQEVFEYCLEDGSLIRATKDHKFMTVDGQMLPIDEIF
301 ERELDLMRVDNLPNVDSGSGETVRFQ/GGGELQKNGIKANFTVRHNVEDGSVQLADHYQQN
361 TPIGDGPVLLPDNHYLSTQTVLSKDPNEKRDHMVLLEFVTAAGITHGMDELYKTSLPETG
421 GHHHHHH*

IntC-mCherry1-IntN-mCherry2
001 MKGSSIKIATRKYLGKQNVYDIGVERDHNFALKNGFIASNCFNGGENLYFQ/GASMVSKGE
061 EDNMAIIKEFMRFKVHMEGSVNGHEFEIEGEGEGRPYEGTQTAKLKVTKGGPLPFAWDIL
121 SPQFMYGSKAYVKHPADIPDYLKLSFPEGFKWERVMNFEDGGVVTVTQDSSLQDGEFIYK
181 VKLRGTNFPSDGPVMQKKTMGWEASSERMYPEDGTCLSYETEILTVEYGLLPIGKIVEKR
241 IECTVYSVDNNGNIYTQPVAQWHDRGEQEVFEYCLEDGSLIRATKDHKFMTVDGQMLPID
301 EIFERELDLMRVDNLPNVDSGSGETVRFQ/GGGELGALKGEIKQRLKLKDGGHYDAEVKTT
361 YKAKKPVQLPGAYNVNIKLDITSHNEDYTIVEQYERAEGRHSTGGMDELYKTSLPETGGH
421 HHHHH*

IntC-mWasabi1-IntN-mWasabi2
001 MKGSSIKIATRKYLGKQNVYDIGVERDHNFALKNGFIASNCFNGGENLYFQ/GASMVSKGE
061 ETTMGVIKPDMKIKLKMEGNVNGHAFVIEGEGEGKPYDGTNTINLEVKEGAPLPFSYDIL
121 TTAFSYGNRAFTKYPDDIPNYFKQSFPEGYSWERTMTFEDKGIVKVKSDISMEEDSFIYE
181 IHLKGENFPPNGPVMQKETTGWDASTERMYVRDGTCLSYETEILTVEYGLLPIGKIVEKR
241 IECTVYSVDNNGNIYTQPVAQWHDRGEQEVFEYCLEDGSLIRATKDHKFMTVDGQMLPID
301 EIFERELDLMRVDNLPNVDSGSGETVRFQ/GGGELGVLKGDVKMKLLLEGGGHHRVDFKTI
361 YRAKKAVKLPDYHFVDHRIEILNHDKDYNKVTVYEIAVARNSTDGMDELYKTSLPETGGH
421 HHHHH*

IntC-YPet1-IntN-YPet2
001 MKGSSIKIATRKYLGKQNVYDIGVERDHNFALKNGFIASNCFNGGENLYFQ/GASMSKGEE
061 LFTGVVPILVELDGDVNGHKFSVSGEGEGDATYGKLTLKLLCTTGKLPVPWPTLVTTLGY
121 GVQCFARYPDHMKQHDFFKSAMPEGYVQERTIFFKDDGNYKTRAEVKFEGDTLVNRIELK
181 GIDFKEDGNILGHKLEYNYNSHNVYITADKGTCLSYETEILTVEYGLLPIGKIVEKRIEC
241 TVYSVDNNGNIYTQPVAQWHDRGEQEVFEYCLEDGSLIRATKDHKFMTVDGQMLPIDEIF
301 ERELDLMRVDNLPNVDSGSGETVRFQ/GGGELQKNGIKANFKIRHNIEDGGVQLADHYQQN
361 TPIGDGPVLLPDNHYLSYQSALFKDPNEKRDHMVLLEFLTAAGITEGMNELYKTSLPETG
421 GHHHHHH*

Supplementary sequence 1 | Amino acid sequences of rtx-FPs. IntC-FP1-IntN-FP2 would form rtx-FP with the intein domain removed and TVMV digestion at the red slash line in vivo, and further give cat-FP in vitro with C-terminal GGHHHHHH removed. The GFP1/GFP2, mCherry1/mCherry2, mWasabi1/mWasabi2, YPet1/YPet2 and IntC/IntN domains were colored as green, pink, cyan, yellow, and purple, respectively. The TVMV (ETVRFQ/G) and TEV (ENLYFQ/G) protease recognition sequences were underlined.

IntC-GFP1-IntN-GFP2-DHFR
001 MKGSSIKIATRKYLGKQNVYDIGVERDHNFALKNGFIASNCFNGGENLYFQ/GASMSKGEE
061 LFTGVVPILVELDGDVNGHKFSVRGEGEGDATIGKLTLKFICTTGKLPVPWPTLVTTLTY
121 GVQCFSRYPDHMKRHDFFKSAMPEGYVQERTISFKDDGKYKTRAVVKFEGDTLVNRIELK
181 GTDFKEDGNILGHKLEYNFNSHNVYITADKGTCLSYETEILTVEYGLLPIGKIVEKRIEC
241 TVYSVDNNGNIYTQPVAQWHDRGEQEVFEYCLEDGSLIRATKDHKFMTVDGQMLPIDEIF
301 ERELDLMRVDNLPNVDSGSGETVRFQ/GGGELQKNGIKANFTVRHNVEDGSVQLADHYQQN
361 TPIGDGPVLLPDNHYLSTQTVLSKDPNEKRDHMVLLEFVTAAGITHGMDELYKTSMISLI
421 AALAVDRVIGMENAMPWNLPADLAWFKRNTLNKPVIMGRHTWESIGRPLPGRKNIILSSQ
481 PGTDDRVTWVKSVDEAIAACGDVPEIMVIGGGRVYEQFLPKAQKLYLTHIDAEVEGDTHF
541 PDYEPDDWESVFSEFHDADAQNSHSYCFEILERRGSLPETGGHHHHHH*

IntC-GFP1-IntN-GFP2-monellin
001 MKGSSIKIATRKYLGKQNVYDIGVERDHNFALKNGFIASNCFNGGENLYFQ/GASMSKGEE
061 LFTGVVPILVELDGDVNGHKFSVRGEGEGDATIGKLTLKFICTTGKLPVPWPTLVTTLTY
121 GVQCFSRYPDHMKRHDFFKSAMPEGYVQERTISFKDDGKYKTRAVVKFEGDTLVNRIELK
181 GTDFKEDGNILGHKLEYNFNSHNVYITADKGTCLSYETEILTVEYGLLPIGKIVEKRIEC
241 TVYSVDNNGNIYTQPVAQWHDRGEQEVFEYCLEDGSLIRATKDHKFMTVDGQMLPIDEIF
301 ERELDLMRVDNLPNVDSGSGETVRFQ/GGGSGGSSGELQKNGIKANFTVRHNVEDGSVQLA
361 DHYQQNTPIGDGPVLLPDNHYLSTQTVLSKDPNEKRDHMVLLEFVTAAGITHGMDELYKG
421 GSGGTSGEWEIIDIGPFTQNLGKFAVDEENKIGQYGRLTFNKVIRPCMKKTIYENEGFRE
481 IKGYEYQLYVRASDKLFRADISEDYKTRGRKLLRFNGPVPPPGSLPETGGHHHHHH*

IntC-GFP1-IntN-GFP2-leptin
001 MKGSSIKIATRKYLGKQNVYDIGVERDHNFALKNGFIASNCFNGGENLYFQ/GASMSKGEE
061 LFTGVVPILVELDGDVNGHKFSVRGEGEGDATIGKLTLKFICTTGKLPVPWPTLVTTLTY
121 GVQCFSRYPDHMKRHDFFKSAMPEGYVQERTISFKDDGKYKTRAVVKFEGDTLVNRIELK
181 GTDFKEDGNILGHKLEYNFNSHNVYITADKGTCLSYETEILTVEYGLLPIGKIVEKRIEC
241 TVYSVDNNGNIYTQPVAQWHDRGEQEVFEYCLEDGSLIRATKDHKFMTVDGQMLPIDEIF
301 ERELDLMRVDNLPNVDSGSGETVRFQ/GGGELQKNGIKANFTVRHNVEDGSVQLADHYQQN
361 TPIGDGPVLLPDNHYLSTQTVLSKDPNEKRDHMVLLEFVTAAGITHGMDELYKTSVPIQK
421 VQDDTKTLIKTIVTRINDISHTQSVSSKQKVTGLDFIPGLHPILTLSKMDQTLAVYQQIL
481 TSMPSRNVIQISNDLENLRDLLHVLAFSKSCHLPEASGLETLDSLGGVLEASGYSTEVVA
541 LSRLQGSLQDMLWQLDLSPGCGSLPETGGHHHHHH*

IntC-GFP1-IntN-GFP2-mCherry
001 MKGSSETVRFQ/GGGKLMEEDNMAIIKEFMRFKVHMEGSVNGHEFEIEGEGEGHPYEGTQT
061 AKLKVTKGGPLPFAWDILSPQFMYGSKAYVKHPADIPDYLKLSFPEGFTWERVMNFEDGG
121 VVTVTQDSSLQDGQFIYKVKLLGINFPSDGPVMQKKTMGWEASTERMYPEDGALKGEINQ
181 RLKLKDGGHYDAEVKTTYKAKKPVQLPGAYNVDIKLDITSHNEDYTIVEQYERAEARHST
241 TSQKNGIKANFTVRHNVEDGSVQLADHYQQNTPIGDGPVLLPDNHYLSTQTVLSKDPNEK
301 RDHMVLLEFVTAAGITHGMDELYKGSLPETGGHHHHHHVDSGSETVRFQ/GGGGSSGELIK
361 IATRKYLGKQNVYDIGVERDHNFALKNGFIASNCFNGGENLYFQ/GASMSKGEELFTGVVP
421 ILVELDGDVNGHKFSVRGEGEGDATIGKLTLKFICTTGKLPVPWPTLVTTLTYGVQCFSR
481 YPDHMKRHDFFKSAMPEGYVQERTISFKDDGKYKTRAVVKFEGDTLVNRIELKGTDFKED
541 GNILGHKLEYNFNSHNVYITADKGTCLSYETEILTVEYGLLPIGKIVEKRIECTVYSVDN
601 NGNIYTQPVAQWHDRGEQEVFEYCLEDGSLIRATKDHKFMTVDGQMLPIDEIFERELDLM
661 RVDNLPN*

Supplementary sequence 2 | Amino acid sequences of rtx-GFP-POI. IntC-GFP1-IntN-GFP2-POI would form rtx-GFP-POI with the intein domain removed and TVMV digestion at the red slash line in vivo, and further give cat-GFP-POI in vitro with C-terminal GGHHHHHH removed. The GFP1, GFP2, DHFR, monellin, leptin, mCherry, and IntC/IntN domains were colored as green, red, orange, yellow, gold, pink, and purple, respectively. The TVMV (ETVRFQ/G) and TEV (ENLYFQ/G) protease recognition sequences were underlined.

IntC-GFP-IntN
001 MGSSMIKIATRKYLGKQNVYDIGVERDHNFALKNGFIASNCFNGGHHHHHHELENLYFQ/G
061 MGSSMSKGEELFTGVVPILVELDGDVNGHKFSVRGEGEGDATIGKLTLKFICTTGKLPVP
121 WPTLVTTLTYGVQCFSRYPDHMKRHDFFKSAMPEGYVQERTISFKDDGKYKTRAVVKFEG
181 DTLVNRIELKGTDFKEDGNILGHKLEYNFNSHNVYITADKQKNGIKANFTVRHNVEDGSV
241 QLADHYQQNTPIGDGPVLLPDNHYLSTQTVLSKDPNEKRDHMVLLEFVTAAGITHGMDEL
301 YKGTCLSYETEILTVEYGLLPIGKIVEKRIECTVYSVDNNGNIYTQPVAQWHDRGEQEVF
361 EYCLEDGSLIRATKDHKFMTVDGQMLPIDEIFERELDLMRVDNLPN*

I-GFP
001 MGSSMSKGEELFTGVVPILVELDGDVNGHKFSVRGEGEGDATIGKLTLKFICTTGKLPVP
061 WPTLVTTLTYGVQCFSRYPDHMKRHDFFKSAMPEGYVQERTISFKDDGKYKTRAVVKFEG
121 DTLVNRIELKGTDFKEDGNILGHKLEYNFNSHNVYITADKQKNGIKANFTVRHNVEDGSV
181 QLADHYQQNTPIGDGPVLLPDNHYLSTQTVLSKDPNEKRDHMVLLEFVTAAGITHGMDEL
181 YKLEHHHHHH*

IntC-GFP-DHFR-IntN
001 MGSSMIKIATRKYLGKQNVYDIGVERDHNFALKNGFIASNCFNGGHHHHHHELENLYFQ/G
061 MGSSMSKGEELFTGVVPILVELDGDVNGHKFSVRGEGEGDATIGKLTLKFICTTGKLPVP
121 WPTLVTTLTYGVQCFSRYPDHMKRHDFFKSAMPEGYVQERTISFKDDGKYKTRAVVKFEG
181 DTLVNRIELKGTDFKEDGNILGHKLEYNFNSHNVYITADKQKNGIKANFTVRHNVEDGSV
241 QLADHYQQNTPIGDGPVLLPDNHYLSTQTVLSKDPNEKRDHMVLLEFVTAAGITHGMDEL
301 YKTSMISLIAALAVDRVIGMENAMPWNLPADLAWFKRNTLNKPVIMGRHTWESIGRPLPG
361 RKNIILSSQPGTDDRVTWVKSVDEAIAACGDVPEIMVIGGGRVYEQFLPKAQKLYLTHID
421 AEVEGDTHFPDYEPDDWESVFSEFHDADAQNSHSYCFEILERRGTCLSYETEILTVEYGL
481 LPIGKIVEKRIECTVYSVDNNGNIYTQPVAQWHDRGEQEVFEYCLEDGSLIRATKDHKFM
541 TVDGQMLPIDEIFERELDLMRVDNLPN*

l-GFP-DHFR
001 MGSSMSKGEELFTGVVPILVELDGDVNGHKFSVRGEGEGDATIGKLTLKFICTTGKLPVP
061 WPTLVTTLTYGVQCFSRYPDHMKRHDFFKSAMPEGYVQERTISFKDDGKYKTRAVVKFEG
121 DTLVNRIELKGTDFKEDGNILGHKLEYNFNSHNVYITADKQKNGIKANFTVRHNVEDGSV
181 QLADHYQQNTPIGDGPVLLPDNHYLSTQTVLSKDPNEKRDHMVLLEFVTAAGITHGMDEL
241 YKTSMISLIAALAVDRVIGMENAMPWNLPADLAWFKRNTLNKPVIMGRHTWESIGRPLPG
301 RKNIILSSQPGTDDRVTWVKSVDEAIAACGDVPEIMVIGGGRVYEQFLPKAQKLYLTHID
361 AEVEGDTHFPDYEPDDWESVFSEFHDADAQNSHSYCFEILERRLEHHHHHH*

IntC-GFP-leptin-IntN
001 MGSSMIKIATRKYLGKQNVYDIGVERDHNFALKNGFIASNCFNGGHHHHHHEL<u>ENLYFQ/G</u>
061 MGSSMSKGEELFTGVVPILVELDGDVNGHKFSVRGEGEGDATIGKLTLKFICTTGKLPVP
121 WPTLVTTLTYGVQCFSRYPDHMKRHDFFKSAMPEGYVQERTISFKDDGKYKTRAVVKFEG
181 DTLVNRIELKGTDFKEDGNILGHKLEYNFNSHNVYITADKQKNGIKANFTVRHNVEDGSV
241 QLADHYQQNTPIGDGPVLLPDNHYLSTQTVLSKDPNEKRDHMVLLEFVTAAGITHGMDEL
301 YKTSVPIQKVQDDTKTLIKTIVTRINDISHTQSVSSKQKVTGLDFIPGLHPILTLSKMDQ
361 TLAVYQQILTSMPSRNVIQISNDLENLRDLLHVLAFSKSCHLPEASGLETLDSLGGVLEA
421 SGYSTEVVALSRLQGSLQDMLWQLDLSPGCGTCLSYETEILTVEYGLLPIGKIVEKRIEC
481 TVYSVDNNGNIYTQPVAQWHDRGEQEVFEYCLEDGSLIRATKDHKFMTVDGQMLPIDEIF
541 ERELDLMRVDNLPN*

l-GFP-leptin
001 MGSSMSKGEELFTGVVPILVELDGDVNGHKFSVRGEGEGDATIGKLTLKFICTTGKLPVP
061 WPTLVTTLTYGVQCFSRYPDHMKRHDFFKSAMPEGYVQERTISFKDDGKYKTRAVVKFEG
121 DTLVNRIELKGTDFKEDGNILGHKLEYNFNSHNVYITADKQKNGIKANFTVRHNVEDGSV
181 QLADHYQQNTPIGDGPVLLPDNHYLSTQTVLSKDPNEKRDHMVLLEFVTAAGITHGMDEL
241 YKTSVPIQKVQDDTKTLIKTIVTRINDISHTQSVSSKQKVTGLDFIPGLHPILTLSKMDQ
301 TLAVYQQILTSMPSRNVIQISNDLENLRDLLHVLAFSKSCHLPEASGLETLDSLGGVLEA
361 SGYSTEVVALSRLQGSLQDMLWQLDLSPGCLEHHHHHH*

IntC-GFP-monellin-IntN
001 MGSSMIKIATRKYLGKQNVYDIGVERDHNFALKNGFIASNCFNGGHHHHHHEL<u>ENLYFQ/G</u>
061 MGSSMSKGEELFTGVVPILVELDGDVNGHKFSVRGEGEGDATIGKLTLKFICTTGKLPVP
121 WPTLVTTLTYGVQCFSRYPDHMKRHDFFKSAMPEGYVQERTISFKDDGKYKTRAVVKFEG
181 DTLVNRIELKGTDFKEDGNILGHKLEYNFNSHNVYITADKQKNGIKANFTVRHNVEDGSV
241 QLADHYQQNTPIGDGPVLLPDNHYLSTQTVLSKDPNEKRDHMVLLEFVTAAGITHGMDEL
301 YKTSGEWEIIDIGPFTQNLGKFAVDEENKIGQYGRLTFNKVIRPCMKKTIYENEGFREIK
361 GYEYQLYVRASDKLFRADISEDYKTRGRKLLRFNGPVPPPGTCLSYETEILTVEYGLLPI
421 GKIVEKRIECTVYSVDNNGNIYTQPVAQWHDRGEQEVFEYCLEDGSLIRATKDHKFMTVD
481 GQMLPIDEIFERELDLMRVDNLPN*

l-GFP-monellin
001 MGSSMSKGEELFTGVVPILVELDGDVNGHKFSVRGEGEGDATIGKLTLKFICTTGKLPVP
061 WPTLVTTLTYGVQCFSRYPDHMKRHDFFKSAMPEGYVQERTISFKDDGKYKTRAVVKFEG
121 DTLVNRIELKGTDFKEDGNILGHKLEYNFNSHNVYITADKQKNGIKANFTVRHNVEDGSV
181 QLADHYQQNTPIGDGPVLLPDNHYLSTQTVLSKDPNEKRDHMVLLEFVTAAGITHGMDEL
241 YKTSGEWEIIDIGPFTQNLGKFAVDEENKIGQYGRLTFNKVIRPCMKKTIYENEGFREIK
301 GYEYQLYVRASDKLFRADISEDYKTRGRKLLRFNGPVPPPLEHHHHHH*

IntC-GFP-mCherry-IntN
001 MGSSMIKIATRKYLGKQNVYDIGVERDHNFALKNGFIASNCFNGGHHHHHHELENLYFQ/G
061 MGSSMSKGEELFTGVVPILVELDGDVNGHKFSVRGEGEGDATIGKLTLKFICTTGKLPVP
121 WPTLVTTLTYGVQCFSRYPDHMKRHDFFKSAMPEGYVQERTISFKDDGKYKTRAVVKFEG
181 DTLVNRIELKGTDFKEDGNILGHKLEYNFNSHNVYITADKQKNGIKANFTVRHNVEDGSV
241 QLADHYQQNTPIGDGPVLLPDNHYLSTQTVLSKDPNEKRDHMVLLEFVTAAGITHGMDEL
301 YKTSMEEDNMAIIKEFMRFKVHMEGSVNGHEFEIEGEGEGHPYEGTQTAKLKVTKGGPLP
361 FAWDILSPQFMYGSKAYVKHPADIPDYLKLSFPEGFTWERVMNFEDGGVVTVTQDSSLQD
421 GQFIYKVKLLGINFPSDGPVMQKKTMGWEASTERMYPEDGALKGEINQRLKLKDGGHYDA
481 EVKTTYKAKKPVQLPGAYNVDIKLDITSHNEDYTIVEQYERAEARHSTGTCLSYETEILT
541 VEYGLLPIGKIVEKRIECTVYSVDNNGNIYTQPVAQWHDRGEQEVFEYCLEDGSLIRATK
601 DHKFMTVDGQMLPIDEIFERELDLMRVDNLPN*

l-GFP-mCherry
001 MGSSMSKGEELFTGVVPILVELDGDVNGHKFSVRGEGEGDATIGKLTLKFICTTGKLPVP
061 WPTLVTTLTYGVQCFSRYPDHMKRHDFFKSAMPEGYVQERTISFKDDGKYKTRAVVKFEG
121 DTLVNRIELKGTDFKEDGNILGHKLEYNFNSHNVYITADKQKNGIKANFTVRHNVEDGSV
181 QLADHYQQNTPIGDGPVLLPDNHYLSTQTVLSKDPNEKRDHMVLLEFVTAAGITHGMDEL
241 YKTSMEEDNMAIIKEFMRFKVHMEGSVNGHEFEIEGEGEGHPYEGTQTAKLKVTKGGPLP
301 FAWDILSPQFMYGSKAYVKHPADIPDYLKLSFPEGFTWERVMNFEDGGVVTVTQDSSLQD
361 GQFIYKVKLLGINFPSDGPVMQKKTMGWEASTERMYPEDGALKGEINQRLKLKDGGHYDA
421 EVKTTYKAKKPVQLPGAYNVDIKLDITSHNEDYTIVEQYERAEARHSTLEHHHHHH*

Supplementary sequence 3 | Amino acid sequences of topological control samples. IntC-GFP(-POI)-IntN would form c-GFP(-POI) with the intein domain removed. The GFP, DHFR, monellin, leptin, mCherry, and IntC/IntN domains were colored as green, orange, yellow, gold, pink, and purple, respectively. The TEV (ENLYFQ/G) protease recognition sequence were was underlined.

IntC1-GFP1-IntN1-IntC2-GFP2-IntN2

001 MKGSSIKIATRKYLGKQNVYDIGVERDHNFALKNGFIASNCFNGGENLYFQ/GASMSKGEE
061 LFTGVVPILVELDGDVNGHKFSVRGEGEGDATIGKLTLKFICTTGKLPVPWPTLVTTLTY
121 GVQCFSRYPDHMKRHDFFKSAMPEGYVQERTISFKDDGKYKTRAVVKFEGDTLVNRIELK
181 GTDFKEDGNILGHKLEYNFNSHNVYITADKGTCLSYETEILTVEYGLLPIGKIVEKRIEC
241 TVYSVDNNGNIYTQPVAQWHDRGEQEVFEYCLEDGSLIRATKDHKFMTVDGQMLPIDEIF
301 ERELDLMRVDNLPNVDSGSGETVRFQ/GGGSGGSSGDHNFALKNGFIASNCFNGGHHHHHH
361 ELQKNGIKANFTVRHNVEDGSVQLADHYQQNTPIGDGPVLLPDNHYLSTQTVLSKDPNEK
421 RDHMVLLEFVTAAGITHGMDELYKGGSGGTSCLSYETEILTVEYGLLPIGKIVEKRIECT
481 VYSVDNNGNIYTQPVAQWHDRGEQEVFEYCLEDGSLIRATKDHKFMTVDGQMLPIDEIFE
541 RELDLMRVDNLPNIKIATRKYLGKQNVYDIGVER*

IntC1-GFP1-IntN1-VidC-GFP2-VidN

001 MKGSSIKIATRKYLGKQNVYDIGVERDHNFALKNGFIASNCFNGGENLYFQ/GASMSKGEE
061 LFTGVVPILVELDGDVNGHKFSVRGEGEGDATIGKLTLKFICTTGKLPVPWPTLVTTLTY
121 GVQCFSRYPDHMKRHDFFKSAMPEGYVQERTISFKDDGKYKTRAVVKFEGDTLVNRIELK
181 GTDFKEDGNILGHKLEYNFNSHNVYITADKGTCLSYETEILTVEYGLLPIGKIVEKRIEC
241 TVYSVDNNGNIYTQPVAQWHDRGEQEVFEYCLEDGSLIRATKDHKFMTVDGQMLPIDEIF
301 ERELDLMRVDNLPNVDSGSGETVRFQ/GGGSGSMIEEKKVTVQELRELYLSGEYTIEIDTP
361 DGYQTIGKWFDKGVLSMVRVATATYETVCAFNHMIQLADNTWVQACELDVGVDIQTAAGI
421 QPVMLVEDTSDAECYDFEVMHPNHRYYGDGIVSHNSGKGGHHHHHHELQKNGIKANFTVR
481 HNVEDGSVQLADHYQQNTPIGDGPVLLPDNHYLSTQTVLSKDPNEKRDHMVLLEFVTAAG
541 ITHGMDELYKGGSGGTSESGCLPKEAVVQIRLTKKG *

Supplementary sequence 4 | Amino acid sequences of the precursors for direct synthesis of cat-GFP in cellulo. IntC1-GFP1-IntN1-IntC2-GFP2-IntN2 would form Npu-cat-GFP in vivo with the intein domains removed. IntC1-GFP1-IntN1-VidC-GFP2-VidN would form VidaL-cat-GFP in vivo with the intein domains removed. GFP1, GFP2, IntC1/IntN1, IntC2/IntN2, and VidC/VidN domains were colored as green, red, purple, blue, and yellow, respectively. The TVMV (ETVRFQ/G) and TEV (ENLYFQ/G) protease recognition sequences were underlined.

IntC1-GFP1-A-IntN1-IntC2-GFP2-A-IntN2 (Design A)

001 MKGSSIKIATRKYLGKQNVYDIGVERDHNFALKNGFIASNCFNGGENLYFQ/GASMSKGEE
061 LFTGVVPILVELDGDVNGHKFSVRGEGEGDATIGKLTLKFICTTGKLPVPWPTLVTTLTY
121 GVQCFSRYPDHMKRHDFFKSAMPEGYVQERTISFKDDGKYKTRAVVKFEGDTLVNRIELK
181 GTDFKEDGNILGHKLEYNFNSHNVYITADKGTCLSYETEILTVEYGLLPIGKIVEKRIEC
241 TVYSVDNNGNIYTQPVAQWHDRGEQEVFEYCLEDGSLIRATKDHKFMTVDGQMLPIDEIF
301 ERELDLMRVDNLPNVDSGSGETVRFQ/GGGSGGSSGDHNFALKNGFIASNCFNGGHHHHHH
361 ELQKNGIKANFTVRHNVEDGSVQLADHYQQNTPIGDGPVLLPDNHYLSTQTVLSKDPNEK
421 RDHMVLLEFVTAAGITHGMDELYKGGSGGTSCLSYETEILTVEYGLLPIGKIVEKRIECT
481 VYSVDNNGNIYTQPVAQWHDRGEQEVFEYCLEDGSLIRATKDHKFMTVDGQMLPIDEIFE
541 RELDLMRVDNLPNIKIATRKYLGKQNVYDIGVER*

IntC1-GFP1-B-IntN1-IntC2-GFP2-B-IntN2 (Design B)
001 MKGSSIKIATRKYLGKQNVYDIGVERDHNFALKNGFIASNCFNGGENLYFQ/GASMSKGEE
061 LFTGVVPILVELDGDVNGGGSGGSDDGKYKTRAVVKFEGDTLVNRIELKGTDFKEDGNIL
121 GHKLEYNFNSHNVYITADKQKNGIKANFTVRHNVEDGSVQLADHYQQNTPIGDGPVLLPD
181 NHYLSTQTVLSKDPNEKRDHMVLLEFVTAAGITHGMDELYKGTCLSYETEILTVEYGLLP
241 IGKIVEKRIECTVYSVDNNGNIYTQPVAQWHDRGEQEVFEYCLEDGSLIRATKDHKFMTV
301 DGQMLPIDEIFERELDLMRVDNLPNVDSGSGETVRFQ/GGGSGGSSGDHNFALKNGFIASN
361 CFNGGHHHHHHELHKFSVRGEGEGDATIGKLTLKFICTTGKLPVPWPTLVTTLTYGVQCF
421 SRYPDHMKRHDFFKSAMPEGYVQERTISFKGGSGGSGGSTSCLSYETEILTVEYGLLPIG
481 KIVEKRIECTVYSVDNNGNIYTQPVAQWHDRGEQEVFEYCLEDGSLIRATKDHKFMTVDG
541 QMLPIDEIFERELDLMRVDNLPNIKIATRKYLGKQNVYDIGVER*

IntC1-GFP1-C-IntN1-IntC2-GFP2-C-IntN2 (Design C)
001 MKGSSIKIATRKYLGKQNVYDIGVERDHNFALKNGFIASNCFNGGENLYFQ/GGGSGGASK
061 DPNEKRDHMVLLEFVTAAGITHGMDELYKGGSGGSMSKGEELFTGVVPILVELDGDVNGH
121 KFSVRGEGEGDATIGGSGGSDTLVNRIELKGTDFKEDGNILGHKLEYNFNSHNVYITADK
181 QKNGIKANFTVRHNVEDGSVQLADHYQQNTPIGDGPVLLPDNHYLSTQTVLSGTCLSYET
241 EILTVEYGLLPIGKIVEKRIECTVYSVDNNGNIYTQPVAQWHDRGEQEVFEYCLEDGSLI
301 RATKDHKFMTVDGQMLPIDEIFERELDLMRVDNLPNVDSGSGETVRFQ/GGGSGGSSGDHN
361 FALKNGFIASNCFNGGHHHHHHELGKLTLKFICTTGKLPVPWPTLVTTLTYGVQCFSRYP
421 DHMKRHDFFKSAMPEGYVQERTISFKDDGKYKTRAVVKFEGGGSGGSGGSTSCLSYETEI
481 LTVEYGLLPIGKIVEKRIECTVYSVDNNGNIYTQPVAQWHDRGEQEVFEYCLEDGSLIRA
541 TKDHKFMTVDGQMLPIDEIFERELDLMRVDNLPNIKIATRKYLGKQNVYDIGVER*

Supplementary sequence 5 | Amino acid sequences of preliminary trials of Design A, B, and C. IntC1-GFP1-(A/B/C)-IntN1-IntC2-GFP2-(A/B/C)-IntN2 were expected to form GFP catenanes in vivo with the intein domain removed and TVMV digestion at the red slash line in vivo. GFP1-(A/B/C), GFP2-(A/B/C), IntC1/IntN1, and IntC2/IntN2 were colored as green, red, purple, and blue. The TVMV (ETVRFQ/G) and TEV (ENLYFQ/G) protease recognition sequences were underlined.

Supplementary Figures

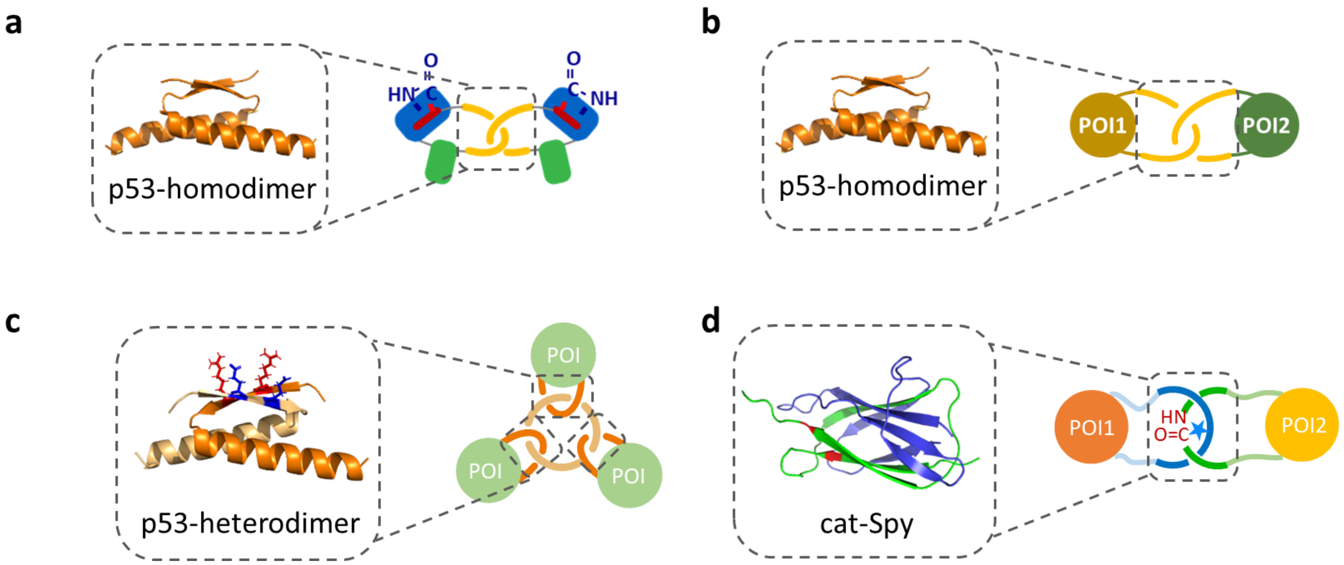

Supplementary Fig. 1 | Multi-domain artificial protein catenanes containing redundant components including relics from entangling templates or scars from chemical ligation. a, p53-homodimer-mediated protein catenane[1]. **b,** p53-homodimer-mediated protein heterocatenane[2]. **c,** p53-heterodimer-mediated protein [n]catenane[3]. **d,** cat-Spy-mediated protein heterocatenane[4].

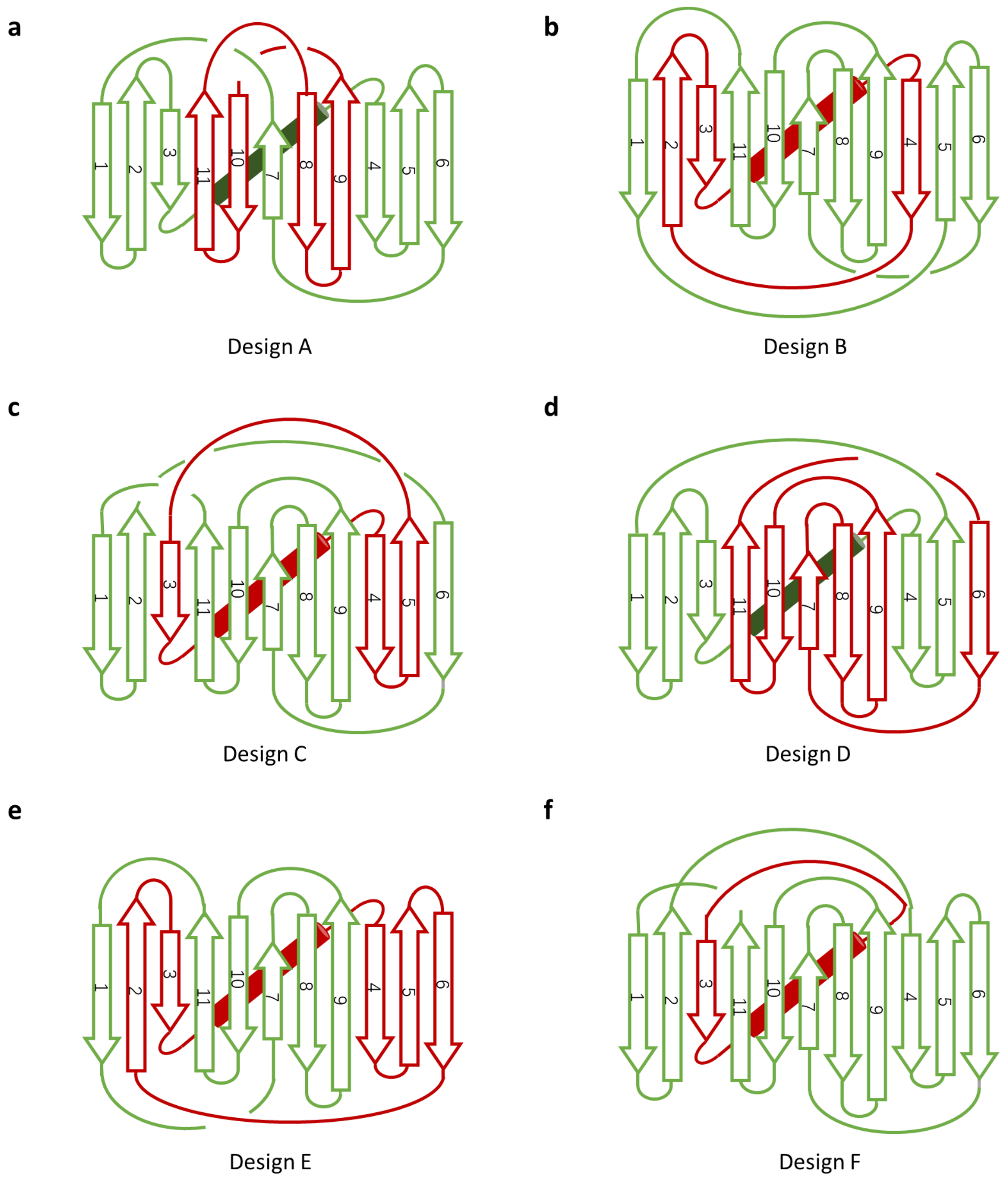
a
Design A
b
Design B
c
Design C
d
Design D
e
Design E
f
Design F

g

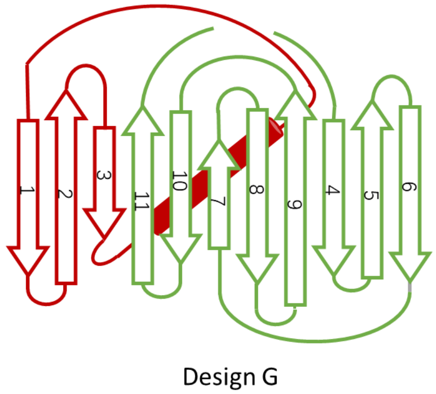

h

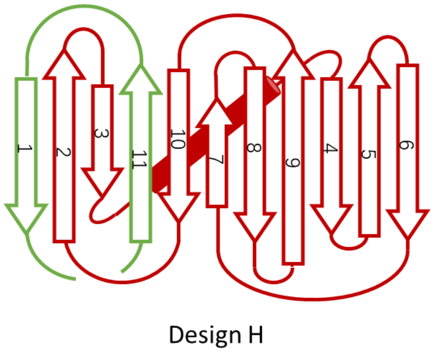

i

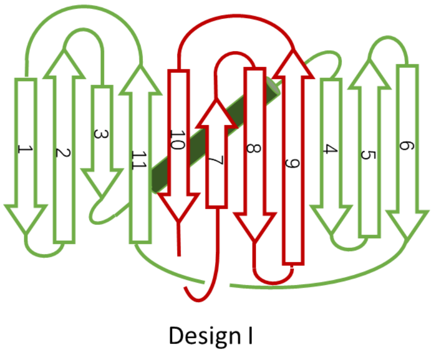

j

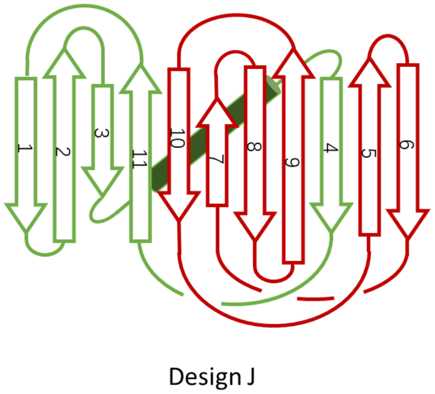

k

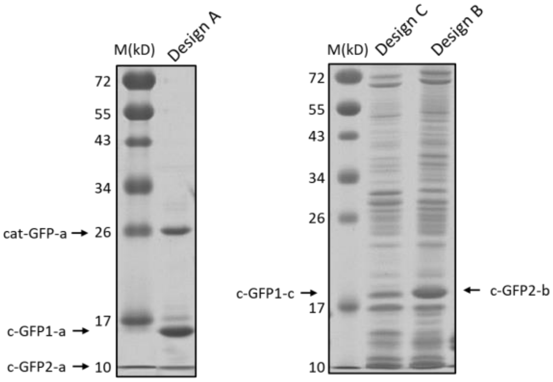

Supplementary Fig. 2 | Topology diagrams of 10 possible catenane designs. a, Design A, Ring1 (green): strand 1-7, Ring2 (red): strand 8-11. **b,** Design B, Ring1 (green): strand 1,5-11 Ring2 (red): strand 2-4. **c,** Design C, Ring1 (green): strand 1,2,6-11 Ring2 (red): strand 3-5. **d,** Design D, Ring1 (green): strand 1-5, Ring2 (red): strand 6-11. **e,** Design E, Ring1 (green): strand 1 and strand 7-11 Ring2 (red): strand 2-6. **f,** Design F, Ring1 (green): strand 1-2 and strand 4-11, Ring2 (red): strand 3. **g,** Design G, Ring1 (green): strand 1-3, Ring2 (red): strand 4-11. **h,** Design H, Ring1 (green): strand 1 and strand 11, Ring2 (red): strand 2-10. **i,** Design I, Ring1 (green): strand 1-6 and strand 11, Ring2 (red): strand 7-10. **j,** Design J, Ring1 (green): strand 1-4 and strand 11, Ring2 (red): strand 5-10. **k,** SDS-PAGE analysis of the products of Design A, Design B, and Design C after Ni-NTA purification by cellular synthesis. Only samples of Design A showed obvious green fluorescence after Ni-NTA purification and exhibited the expected molecular weight of GFP catenane (~30 kDa) in SDS-PAGE. Source data for SDS-PAGE are provided in the Source Data for Supplementary Figures.

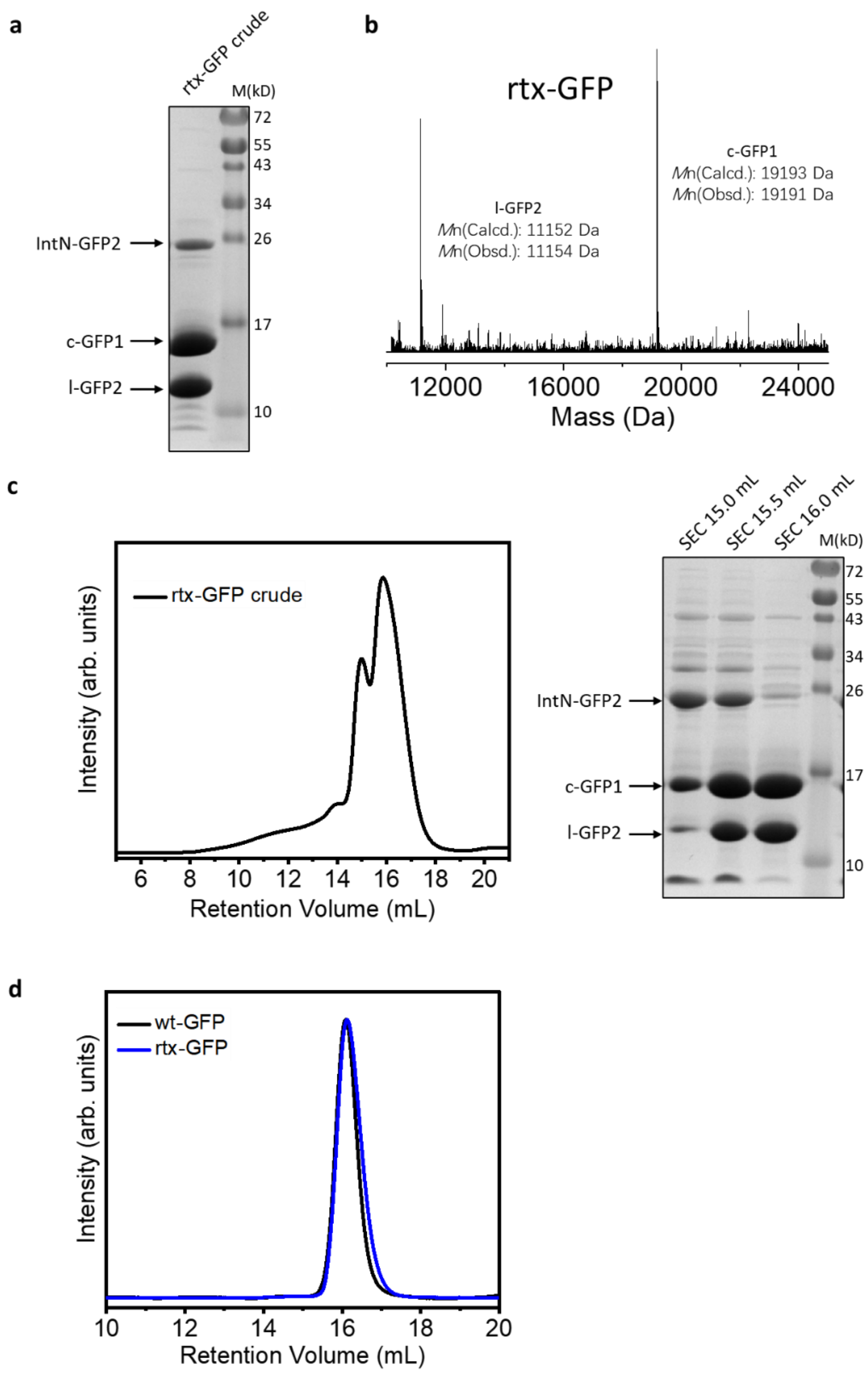

Supplementary Fig. 3 | Purification of rtx-GFP. a, SDS-PAGE of crude products after Ni-NTA affinity purification, showing three bands. IntN-GFP2 was the incomplete TVMV digestion products. **b,** LC-MS of rtx-GFP. The LC-MS spectrum confirms the identity of rtx-GFP with the correct observed mass of 11154 Da versus the calculated value of 11152 Da for c-GFP1 as well as the observed mass of 19191 Da versus the calculated value of 19193 Da for l-GFP2. **c,** SEC trace of the crude product and the corresponding SDS-PAGE showing the product distribution. The protein samples at the second peak on SEC trace (~16 mL) were the purified rtx-GFP, which were taken as reactants for the subsequent cyclization. **d,** SEC overlay of purified rtx-GFP (blue) and wt-GFP (black). Source data for SDS-PAGE are provided in the Source Data for Supplementary Figures.

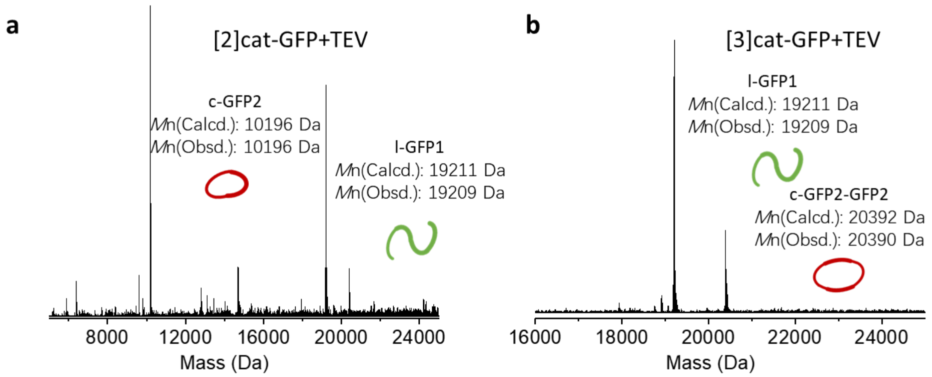

Supplementary Fig. 4 | LC-MS spectra of TEV digestion products of [2]cat-GFP and [3]cat-GFP. a, l-GFP1 and c-GFP2 produced by proteolytic digestion of [2]cat-GFP by TEV protease. **b,** l-GFP1 and c-GFP2-GFP2 produced by proteolytic digestion of [3]cat-GFP by TEV protease.

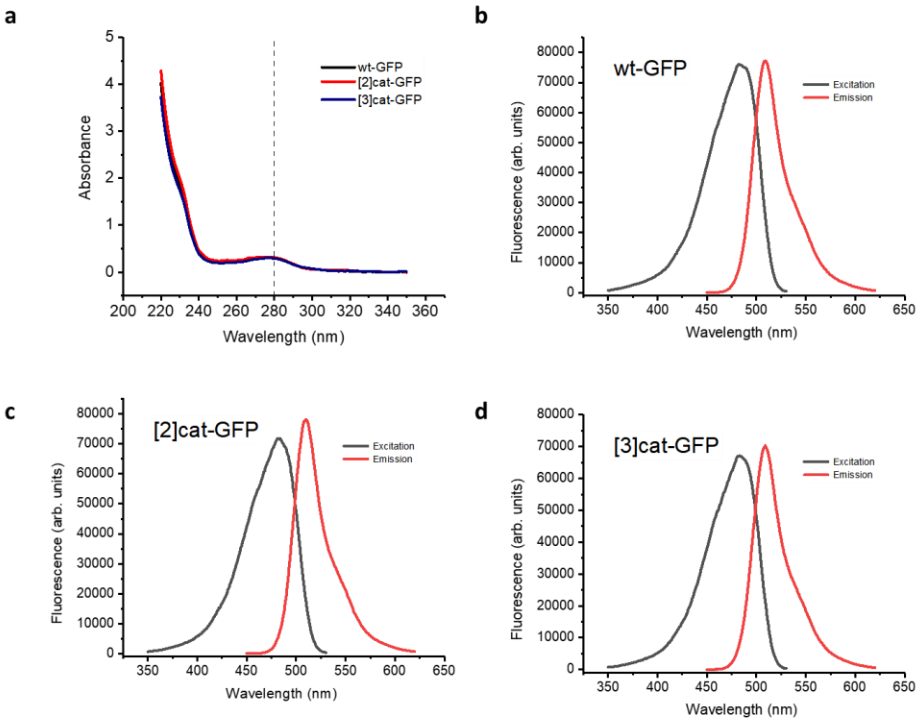

Supplementary Fig. 5 | UV absorption spectra and fluorescence spectra of wt-GFP (l-GFP), [2]cat-GFP, and [3]cat-GFP. a, Overlay of UV absorption spectra of wt-GFP (black), [2]cat-GFP (red), and [3]cat-GFP (blue). The absorbance at 280 nm showed the concentration of wt-GFP, [2]cat-GFP, and [3]cat-GFP were approximately 10 µM, 10 µM, and 5 µM, respectively. All samples were then diluted tenfold before fluorescence spectra measurement. **b,** Emission spectrum (red) and excitation spectrum (black) of wt-GFP. **c,** Emission spectrum (red) and excitation spectrum (black) of [2]cat-GFP. **d,** Emission spectrum (red) and excitation spectrum (black) of [3]cat-GFP.

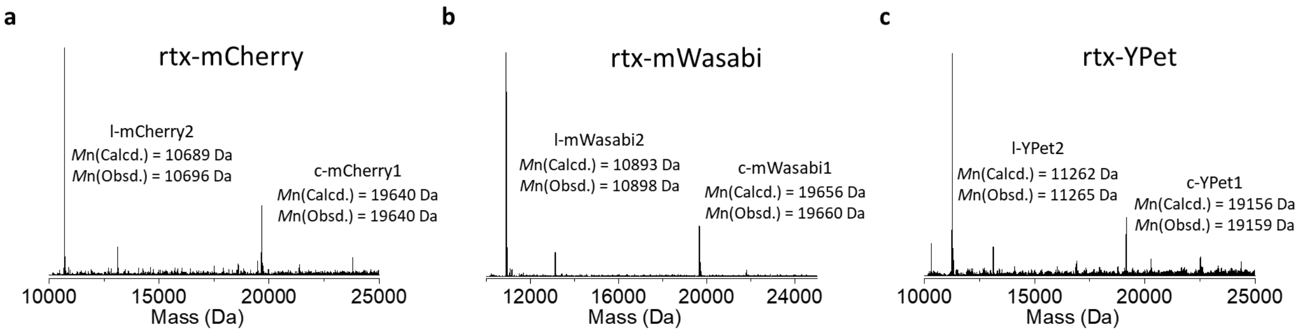

Supplementary Fig. 6 | LC-MS spectra of rxt-FPs. a, rtx-mCherry. **b,** rtx-mWasabi. **c,** rtx-YPet.

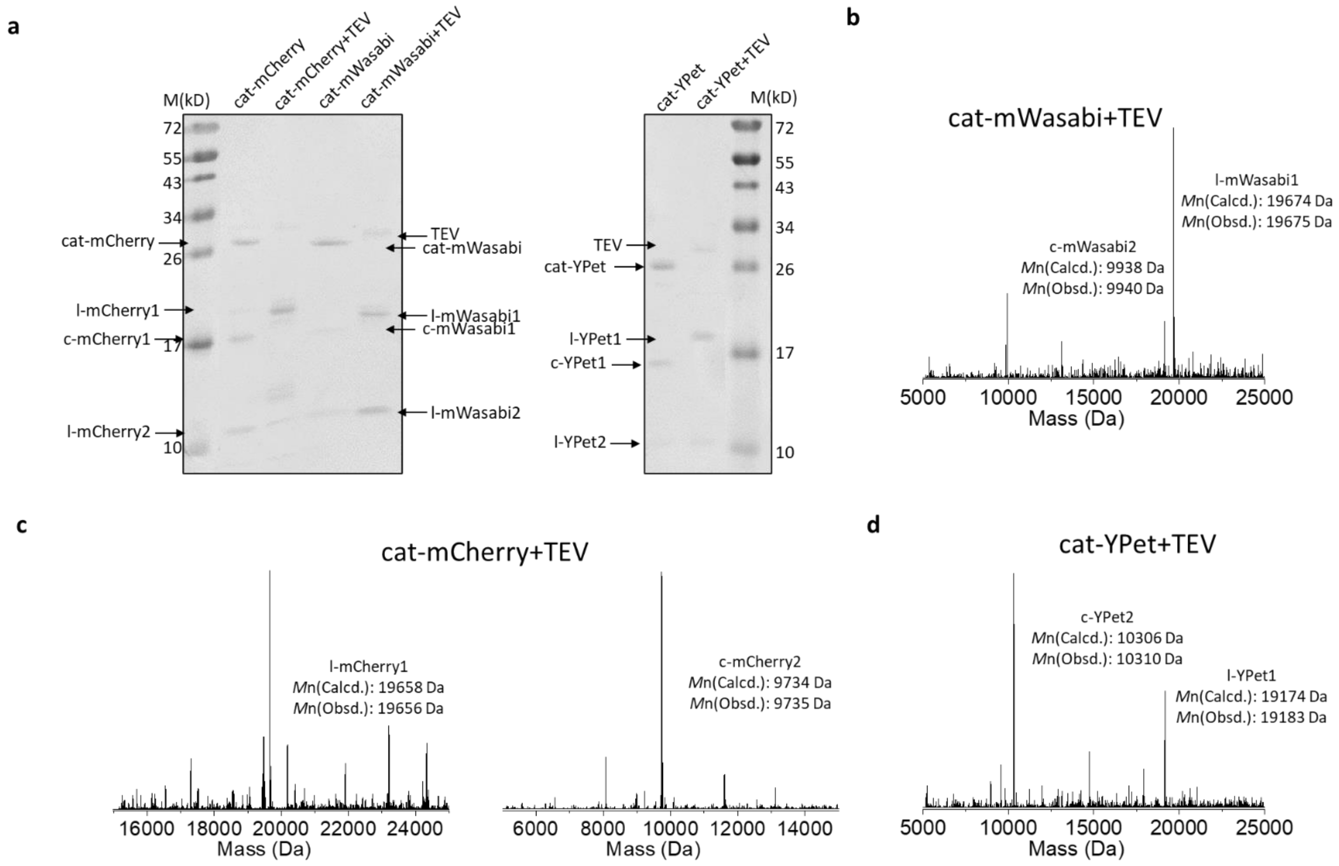

Supplementary Fig. 7 | LC-MS spectra and SDS-PAGE analysis of TEV digestion products. a, SDS-PAGE analysis of cat-FPs and their TEV digestion products. **b,** LC-MS spectra of cat-mWasabi after proteolytic digestion by TEV protease. **c,** LC-MS spectra of cat-mCherry after proteolytic digestion by TEV protease. **d,** LC-MS spectra of cat-YPet after proteolytic digestion by TEV protease. Source data for SDS-PAGE are provided in the Source Data for Supplementary Figures.

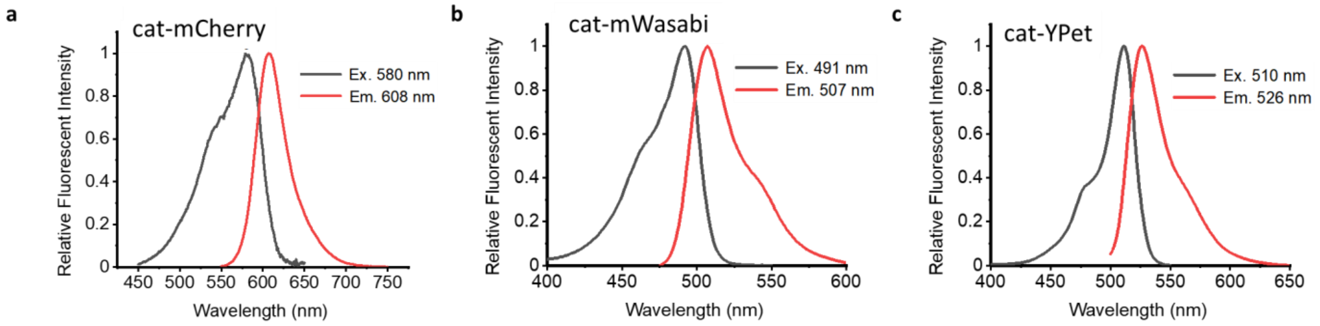

Supplementary Fig. 8 | Emission spectra (red) and excitation spectra (black) of cat-FPs. a, cat-mCherry. **b,** cat-mWasabi. **c,** cat-YPet. They are all consistent with their corresponding reported fluorescence spectra on the FP base (https://www.fpbase.org/).

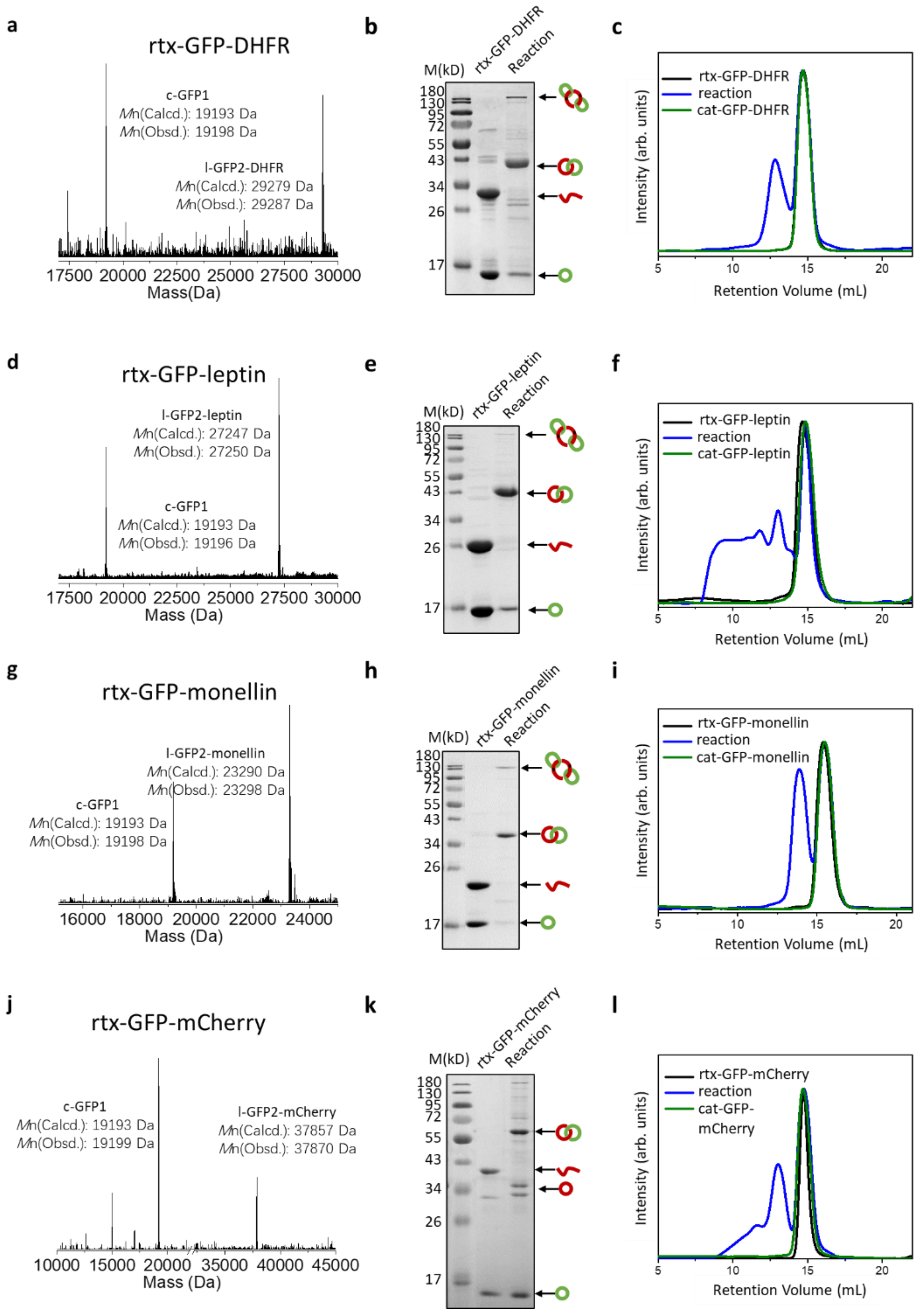

Supplementary Fig. 9 | Characterization of rtx-GFP-POIs and its conversion into cat-GFP-POIs. a, LC-MS spectrum of rtx-GFP-DHFR. **b,** SDS-PAGE analysis of the reaction forming cat-GFP-DHFR. **c,** SEC trace of rtx-GFP-DHFR (black), the reaction forming cat-GFP-DHFR (blue), and the purified cat-GFP-DHFR (green). **d,** LC-MS spectrum of rtx-GFP-leptin. **e,** SDS-PAGE analysis of the reaction forming cat-GFP-leptin. **f,** SEC trace of rtx-GFP-leptin (black), the reaction forming cat-GFP-leptin (blue), and the purified cat-GFP-leptin (green). **g,** LC-MS spectrum of rtx-GFP-monellin. **h,** SDS-PAGE analysis of the reaction forming cat-GFP-monellin. **i,** SEC trace of rtx-GFP-monellin (black), the reaction forming cat-GFP-monellin (blue), and the purified cat-GFP-monellin (green). **j,** LC-MS spectrum of rtx-GFP-mCherry. **k,** SDS-PAGE analysis of the reaction forming cat-mCherry. **l,** SEC trace of rtx-GFP-mCherry (black), the reaction forming cat-GFP-mCherry (blue), and the purified cat-GFP-mCherry (green). Source data for SDS-PAGE are provided in the Source Data for Supplementary Figures.

Note: The selectivity of rtx-GFP-mCherry was lower than other rtx-GFP-POIs. An obvious band for c-GFP2 was observed.

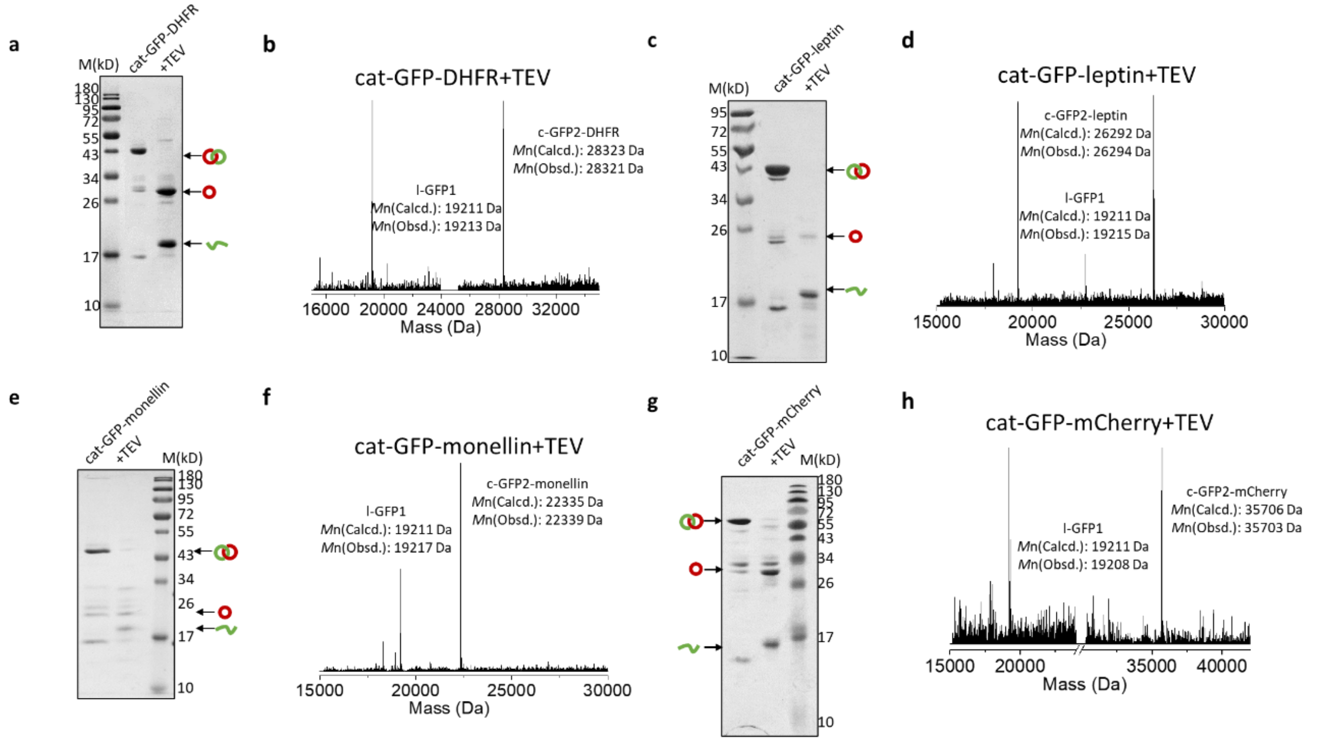

Supplementary Fig. 10 | LC-MS spectra of TEV digestion products of cat-GFP-POIs. a, SDS-PAGE analysis of the TEV protease digestion products of cat-GFP-DHFR. **b,** LC-MS spectrum of the TEV protease digestion products of cat-GFP-DHFR. **c,** SDS-PAGE analysis of the TEV protease digestion products of cat-GFP-leptin. **d,** LC-MS spectrum of the TEV protease digestion products of cat-GFP-leptin. **e,** SDS-PAGE analysis of the TEV protease digestion products of cat-GFP-monellin. **f,** LC-MS spectrum of the TEV protease digestion products of cat-GFP-monellin. **g,** SDS-PAGE analysis of the TEV protease digestion products of cat-GFP-mCherry. **h,** LC-MS spectrum of the TEV protease digestion products of cat-GFP-mCherry. Source data for SDS-PAGE are provided in the Source Data for Supplementary Figures.

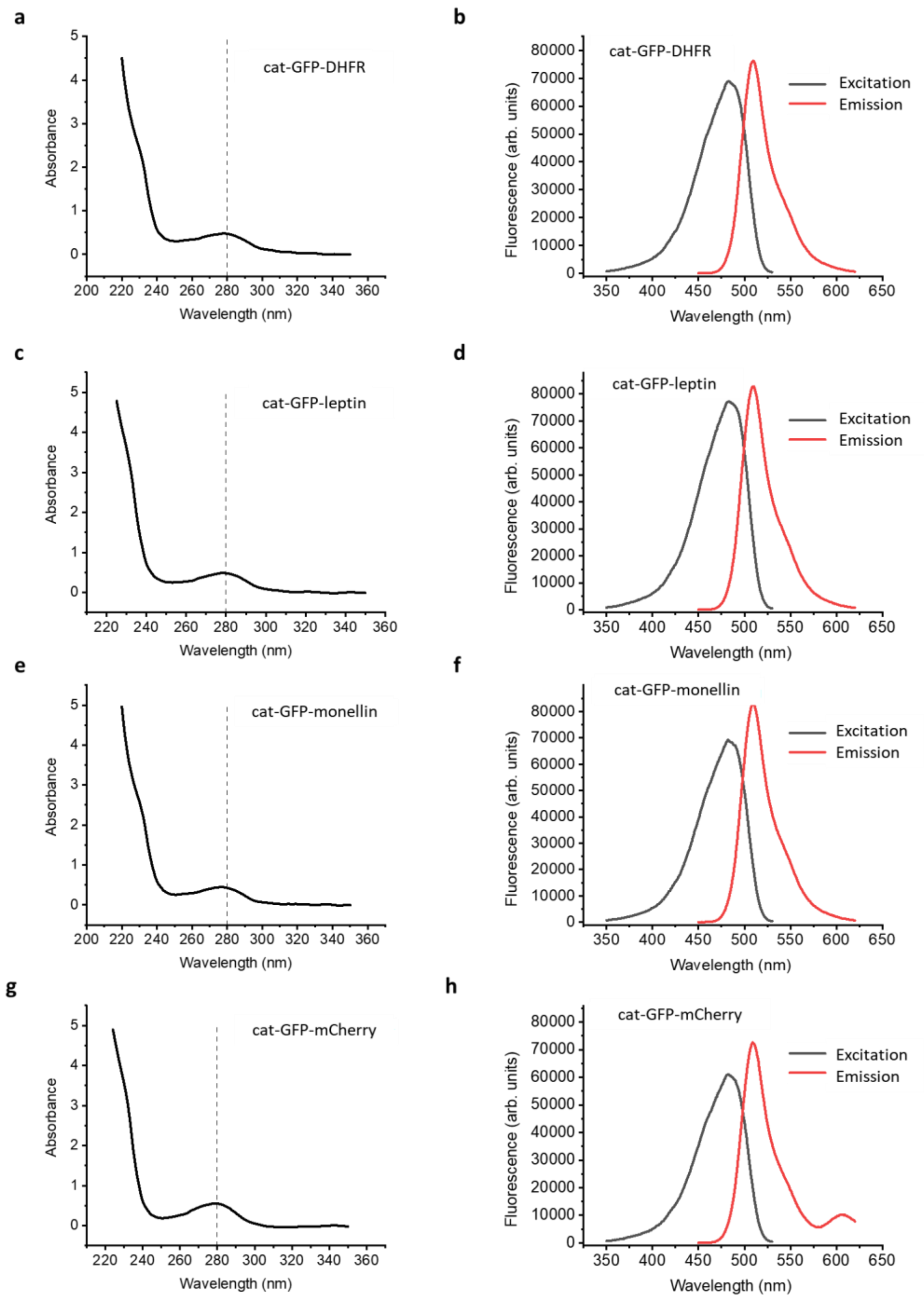

Supplementary Fig. 11 | Fluorescence spectra of cat-GFP-POIs. a, UV absorption spectrum of cat-GFP-DHFR. **b,** Emission spectrum (red) and excitation spectrum (black) of cat-GFP-DHFR. **c,** UV absorption spectrum of cat-GFP-leptin. **d,** Emission spectrum (red) and excitation spectrum (black) of cat-GFP-leptin. **e,** UV absorption spectrum of cat-GFP-monellin. **f,** Emission spectrum (red) and excitation spectrum (black) of cat-GFP-monellin. **g,** UV absorption spectrum of cat-GFP-mCherry. **h,** Emission spectrum (red) and excitation spectrum (black) of cat-GFP-mCherry, the peak at 610 nm is the emission peak of mCherry. The cat-GFP-POI samples were adjusted to ~10 μM as determined by the absorbance at 280 nm, and then further diluted tenfold before fluorescence spectra measurement.

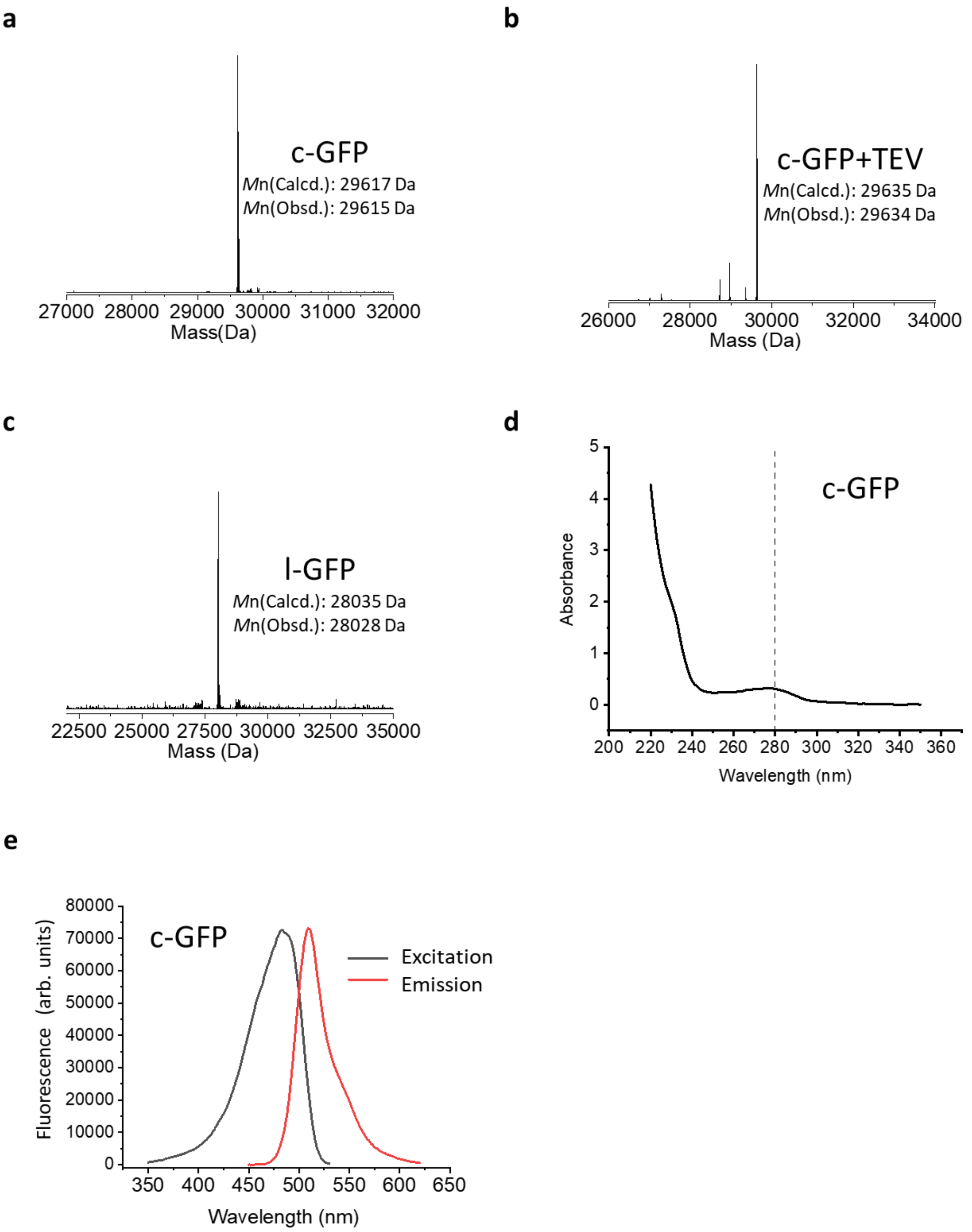

Supplementary Fig. 12 | Characterization of topological controls for the single-domain GFP. a, LC-MS spectrum of c-GFP. **b,** LC-MS spectrum of the TEV protease digestion products of c-GFP. **c,** LC-MS spectrum of l-GFP. **d,** UV absorption spectrum of c-GFP. **e,** Emission spectrum (red) and excitation spectrum (black) of c-GFP. The c-GFP sample was adjusted to ~10 µM as determined by the absorbance at 280 nm, and then further diluted tenfold before fluorescence spectra measurement.

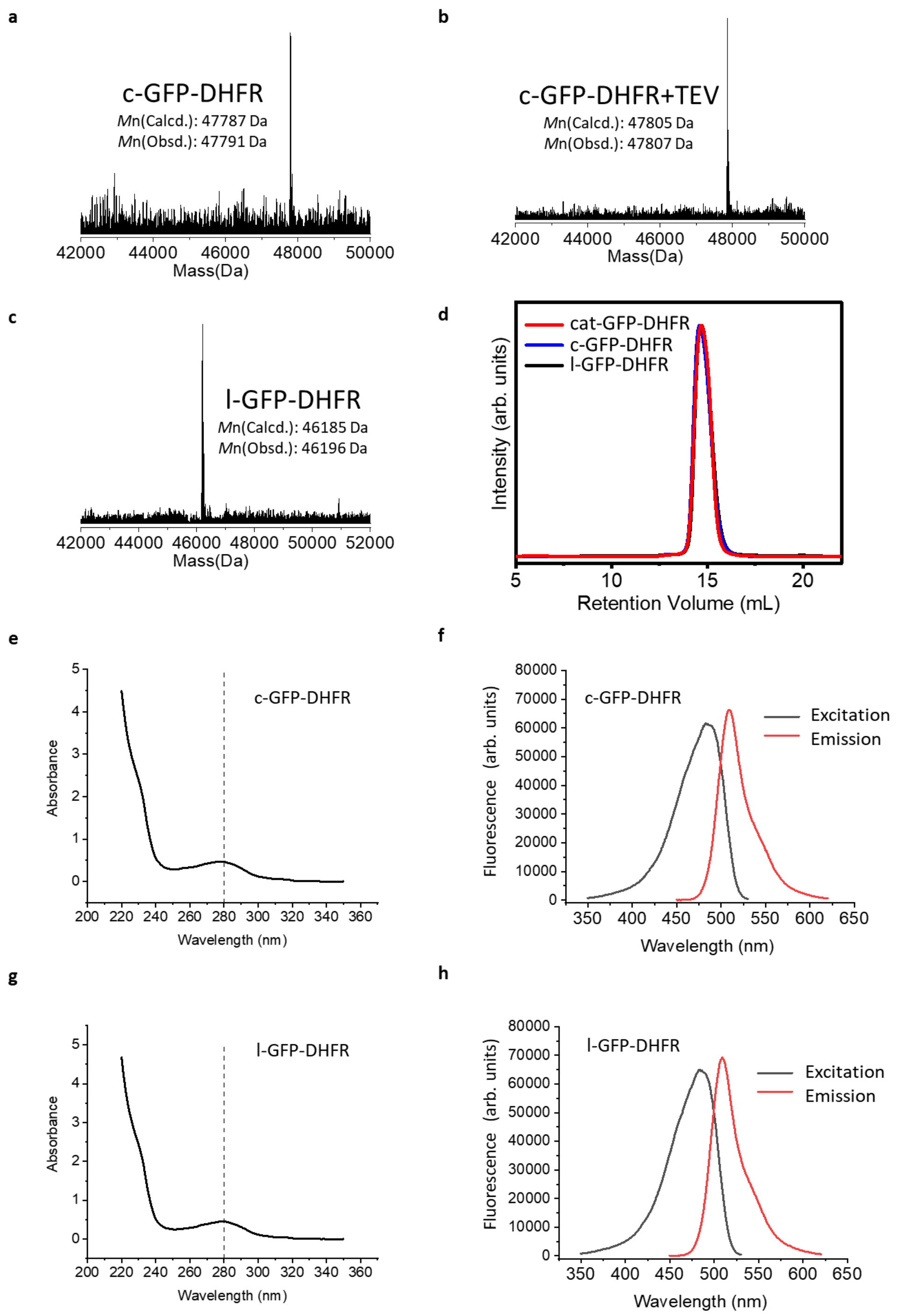

Supplementary Fig. 13 | Characterization of topological controls containing DHFR. a, LC-MS spectrum of c-GFP-DHFR. **b,** LC-MS spectrum of the TEV protease digestion products of c-GFP-DHFR. **c,** LC-MS spectrum of l-GFP-DHFR. **d,** SEC overlay of cat-GFP-DHFR (red), c-GFP-DHFR (blue), and l-GFP-DHFR (black). **e,** UV absorption spectrum of c-GFP-DHFR. **f,** Emission spectrum (red) and excitation spectrum (black) of c-GFP-DHFR. **g,** UV absorption spectrum of l-GFP-DHFR. **h,** Emission spectrum (red) and excitation spectrum (black) of l-GFP-DHFR. The c-GFP-DHFR and l-GFP-DHFR samples were adjusted to ~10 µM as determined by the absorbance at 280 nm, and then further diluted tenfold before fluorescence spectra measurement.

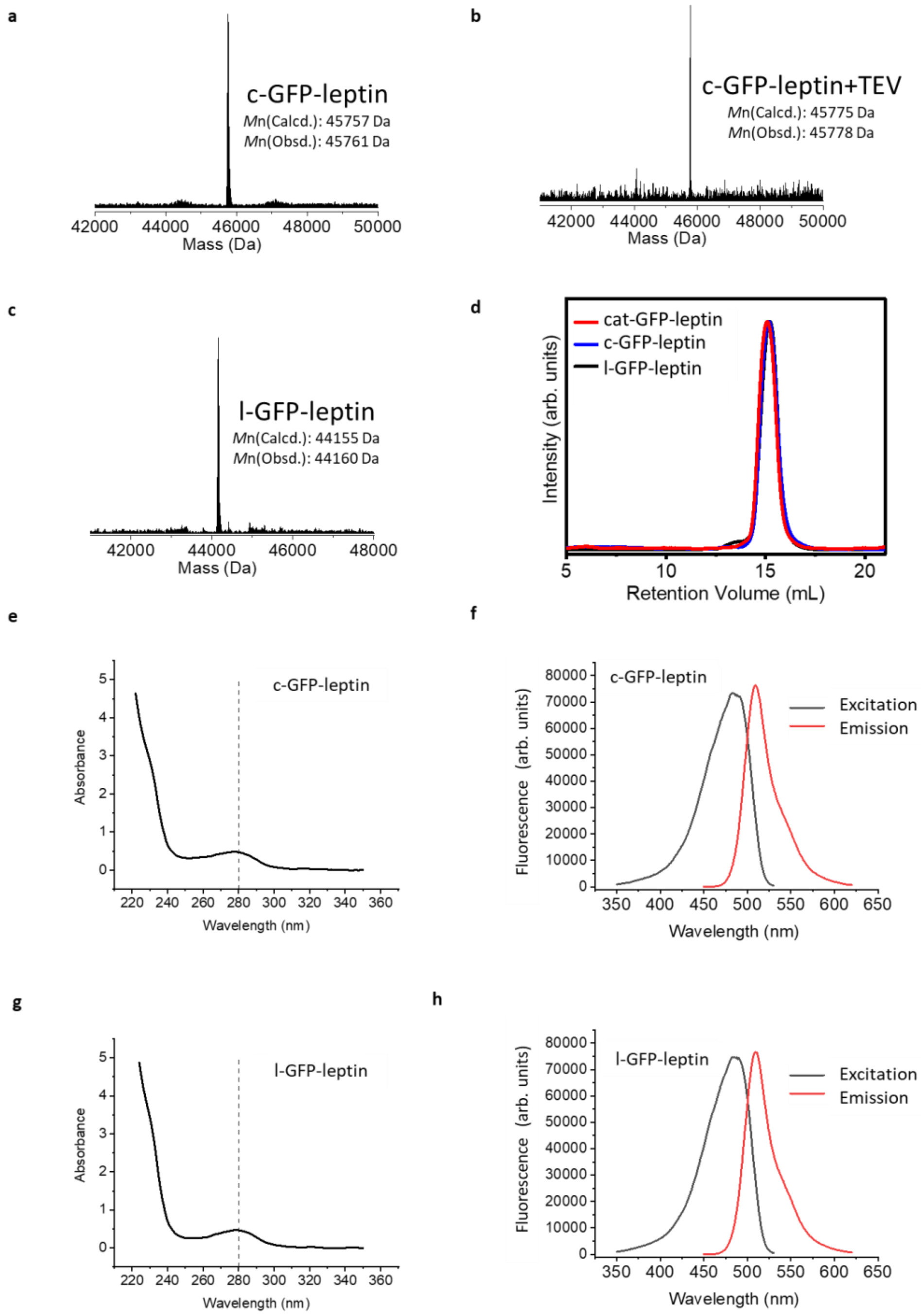

Supplementary Fig. 14 | Characterization of topological controls containing leptin. a, LC-MS spectrum of c-GFP-leptin. **b,** LC-MS spectrum of the TEV protease digestion products of c-GFP-leptin. **c,** LC-MS spectrum of l-GFP-leptin. **d,** SEC overlay of cat-GFP-leptin (red), c-GFP-leptin (blue), and l-GFP-leptin (black). **e,** UV absorption spectrum of c-GFP-leptin. **f,** Emission spectrum (red) and excitation spectrum (black) of c-GFP-leptin. **g,** UV absorption spectrum of l-GFP-leptin. **h,** Emission spectrum (red) and excitation spectrum (black) of l-GFP-leptin. The c-GFP-leptin and l-GFP-leptin samples were adjusted to ~10 μM as determined by the absorbance at 280 nm, and then further diluted tenfold before fluorescence spectra measurement.

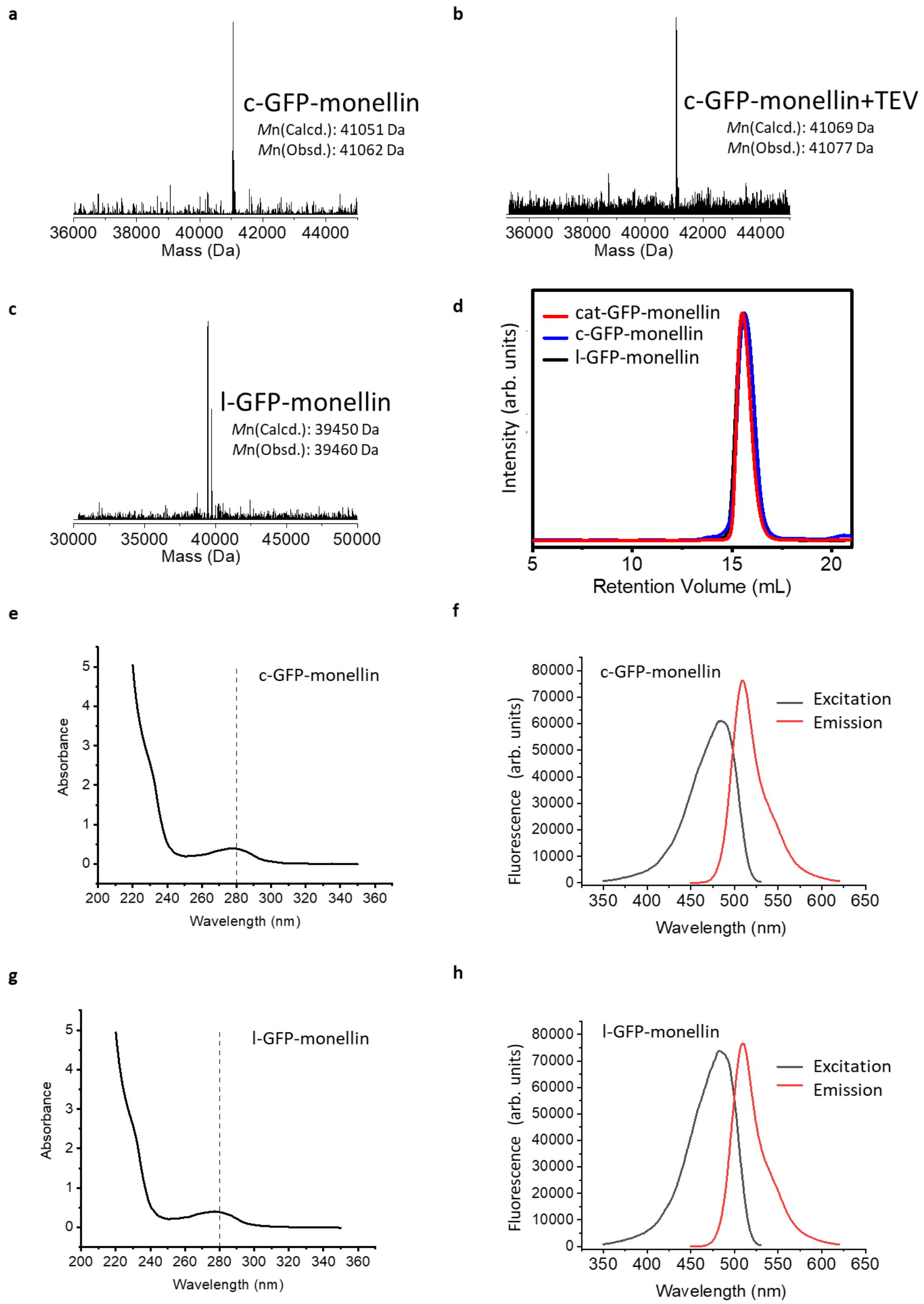

Supplementary Fig. 15 | Characterization of topological controls containing monellin. a, LC-MS spectrum of c-GFP-monellin. **b,** LC-MS spectrum of the TEV protease digestion products of c-GFP-monellin by TEV protease. **c,** LC-MS spectrum of l-GFP-monellin. **d,** SEC overlay of cat-GFP-monellin (red), c-GFP-monellin (blue), and l-GFP-monellin (black). **e,** UV absorption spectrum of c-GFP-monellin. **f,** Emission spectrum (red) and excitation spectrum (black) of c-GFP-monellin. **g,** UV absorption spectra of l-GFP-monellin. **h,** Emission spectrum (red) and excitation spectrum (black) of l-GFP-monellin. The c-GFP-monellin and l-GFP-monellin samples were adjusted to ~10 μM as determined by the absorbance at 280 nm, and then further diluted tenfold before fluorescence spectra measurement.

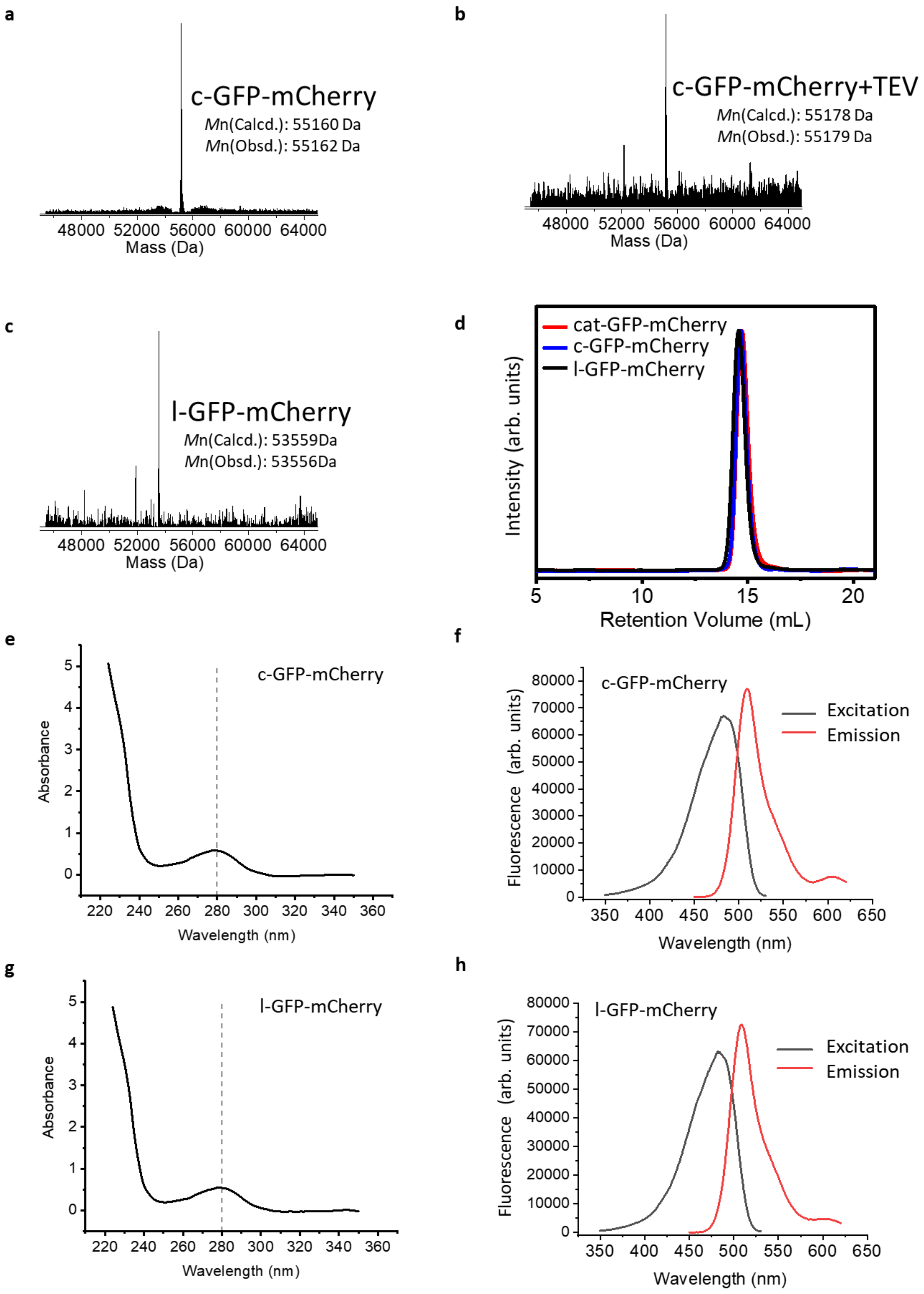

Supplementary Fig. 16 | Characterization of topological controls containing mCherry. a, LC-MS spectrum of c-GFP-mCherry. **b,** LC-MS spectrum of the TEV protease digestion products of c-GFP-mCherry. **c,** LC-MS spectrum of l-GFP-mCherry. **d,** SEC overlay of cat-GFP-mCherry (red), c-GFP-mCherry (blue), and l-GFP-mCherry (black). **e,** UV absorption spectrum of c-GFP-mCherry. **f,** Emission spectrum (red) and excitation spectrum (black) of c-GFP-mCherry. **g,** UV absorption spectrum of l-GFP-mCherry. **h,** Emission spectrum (red) and excitation spectrum (black) of l-GFP-mCherry, the peak at 610 nm is the emission peak of mCherry. The c-GFP-mCherry and l-GFP-mCherry samples were adjusted to ~10 µM as determined by the absorbance at 280 nm, and then further diluted tenfold before fluorescence spectra measurement.

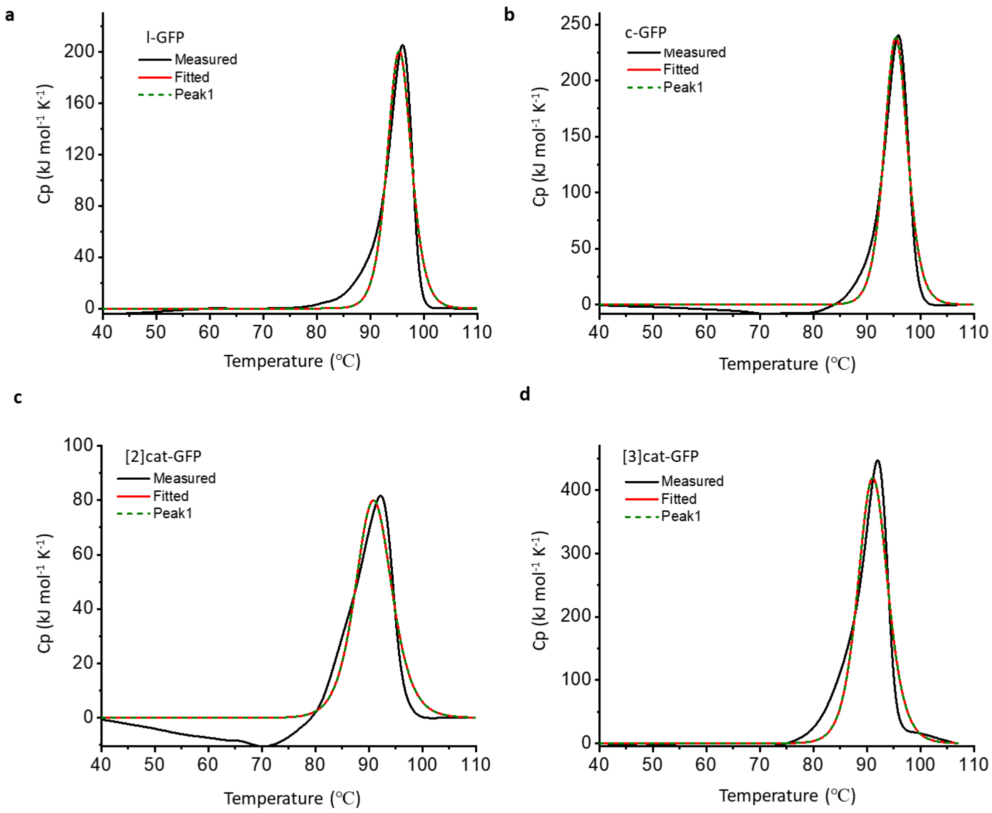

Supplementary Fig. 17 | DSC thermograms of a, l-GFP. **b,** c-GFP. **c,** [2]cat-GFP. **d,** [3]cat-GFP. The curves were fitted using MicroCal PEAQ-DSC Software (Malvern Panalytical Ltd.). The measured curves are shown by black solid lines, the fitted curves are shown by red solid lines, and the fitted curves for peak1 are shown by green dashed lines.

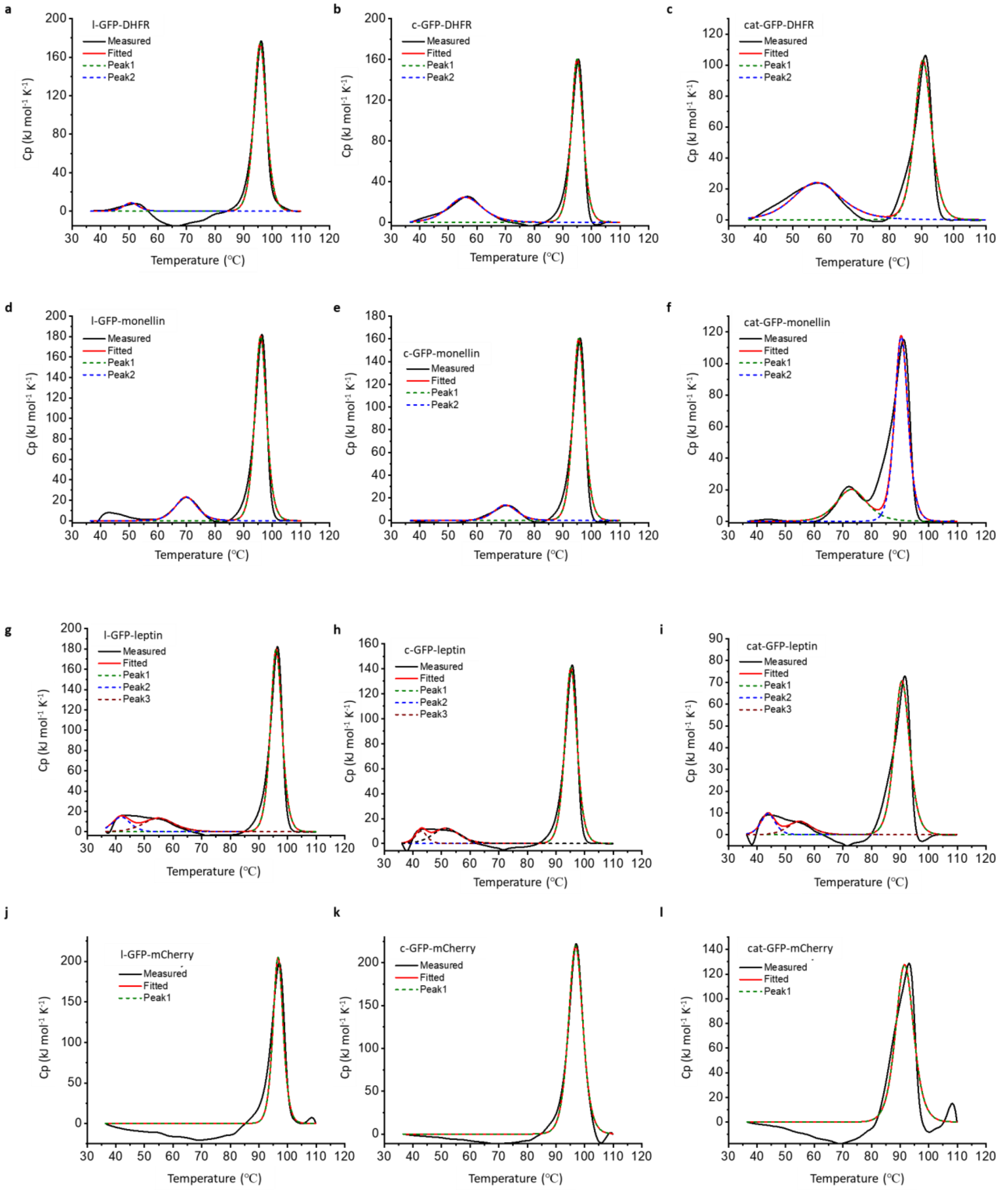

Supplementary Fig. 18 | DSC thermograms of a, l-GFP-DHFR. **b,** c-GFP-DHFR. **c,** cat-GFP-DHFR. **d,** l-GFP-monellin. **e,** c-GFP-monellin. **f,** cat-GFP-monellin. **g,** l-GFP-leptin. **h,** c-GFP-leptin. **i,** cat-GFP-leptin. **g,** l-GFP-mCherry. **h,** c-GFP-mCherry. **i,** cat-GFP-mCherry. T_m values were summerized in **Supplementary Table 2.** The measured curves are shown by black solid lines, the fitted curves are shown by red solid lines, the fitted curves for peak1 are shown by green dashed lines, the fitted curves for peak2 are shown by blue dashed lines, and the fitted curves for peak3 are shown by brown dashed lines.

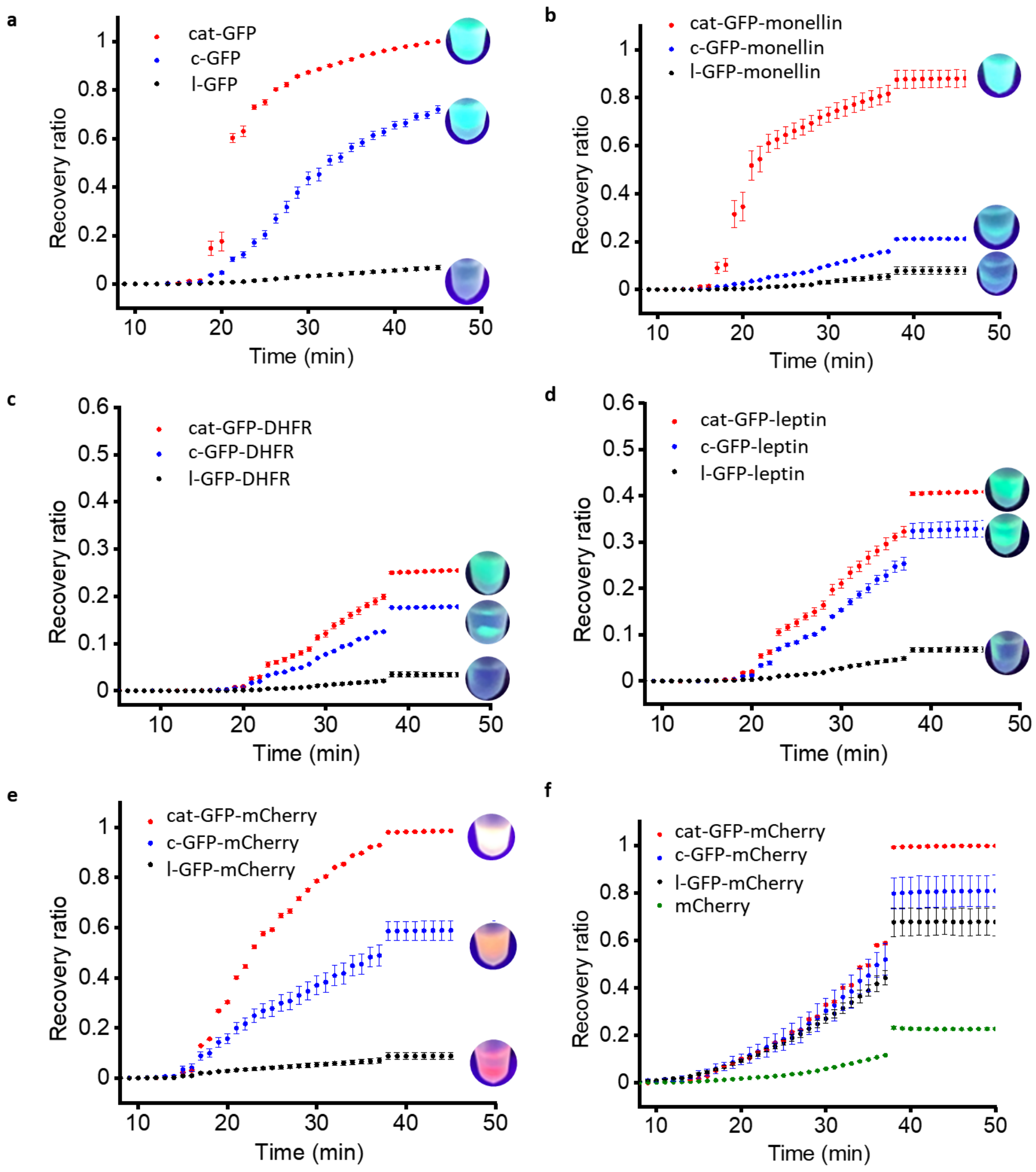

Supplementary Fig. 19 | Fluorescence recovery profiles of protein catenanes and the topological controls. a, Fluorescence recovery curves monitored at 520 nm channel and photos of cat-GFP (red), c-GFP (blue), and l-GFP (black) after boiling and cooling using slow recovery method that cooled down to 25 °C. **b,** Fluorescence recovery curves monitored at 520 nm channel and photos of cat-GFP-monellin (red), c-GFP-monellin (blue), and l-GFP-monellin (black) after boiling and cooling using fast recovery method that cooled down to 5 °C. **c,** Fluorescence recovery curves monitored at 520 nm channel and photos of cat-GFP-DHFR (red), c-GFP-DHFR (blue), and l-GFP-DHFR (black) after boiling and cooling using fast recovery method that cooled down to 5 °C. **d,** Fluorescence recovery curves monitored at 520 nm channel and photos of cat-GFP-leptin (red), c-GFP-leptin (blue), and l-GFP-leptin (black) after boiling and cooling. **e,** Fluorescence recovery curves monitored at 520 nm channel and photos of cat-GFP-mCherry (red), c-GFP-mCherry (blue), and l-GFP-mCherry (black) after boiling and cooling using fast recovery method that cooled down to 5 °C. **f,** Fluorescence recovery curves monitored at 610 nm channel of cat-GFP-mCherry (red), c-GFP-mCherry (blue), and l-GFP-mCherry (black) mCherry (green) after boiling and cooling using fast recovery method that cooled down to 5 °C. Data points are presented as mean values of triplicates (n = 3 independent experiments) with ± standard deviation shown as error bars.

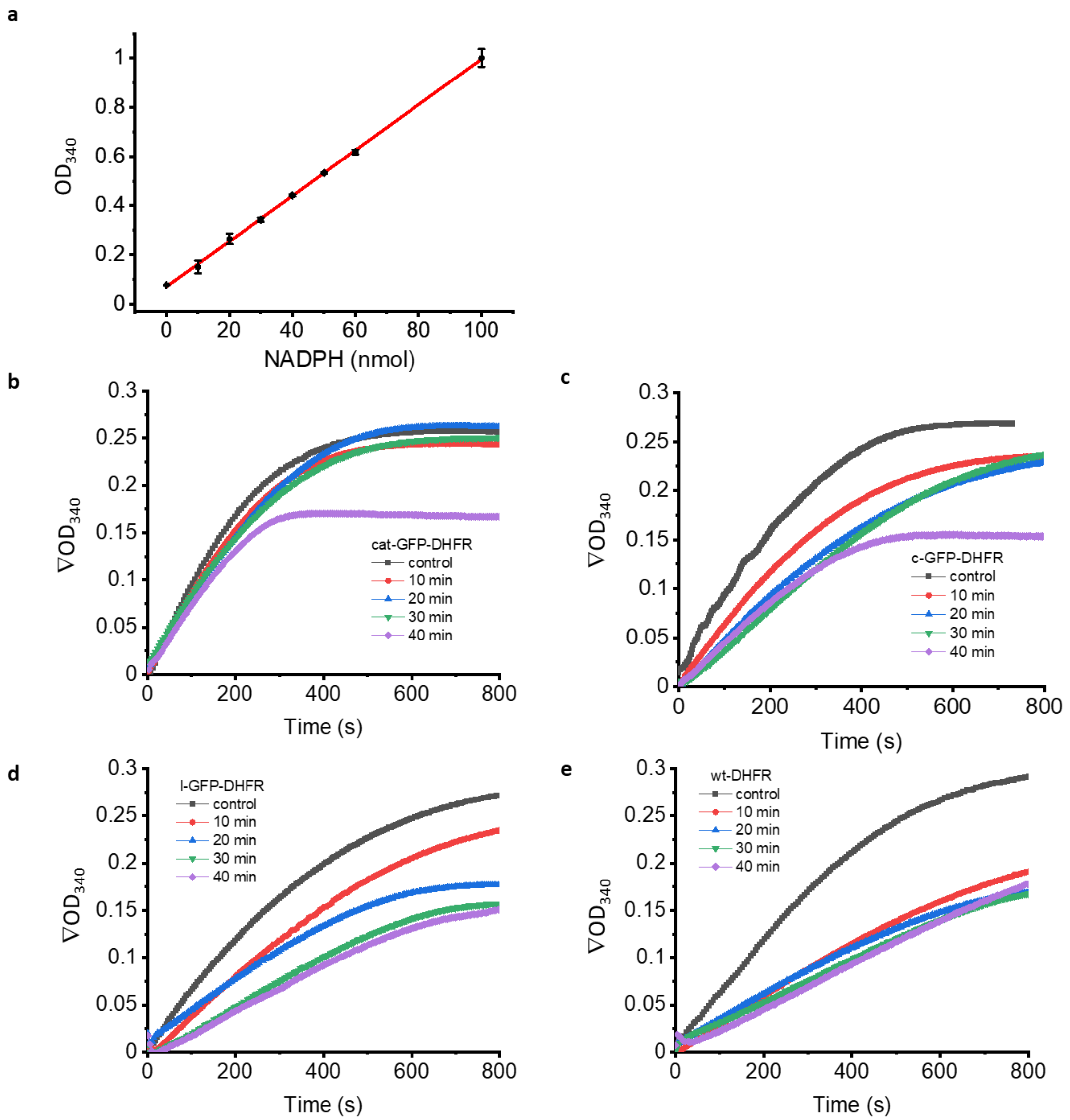

Supplementary Fig. 20 | DHFR catalytic activity assay after incubation at 50 °C. a, the absorbance of NADPH solution with gradient concentration at 340 nm was measured, and linearly fitted to be used as the standard curve: $y = 0.00927\ x + 0.06906$ (x, the amount of NADPH; y, the absorbance at 340 nm). Data points are presented as mean values of triplicates (n = 3 independent experiments) with ± standard deviation shown as error bars. **b,** Kinetic activities of cat-GFP-DHFR with various heating time at 50 °C. **c,** Kinetic activities of c-GFP-DHFR with various heating time at 50 °C. **d,** Kinetic activities of l-GFP-DHFR with various heating time at 50 °C. **e,** Kinetic activities of wt-DHFR with various heating time at 50 °C. The linear region of kinetic activity plots indicated the rate of the catalytic process.

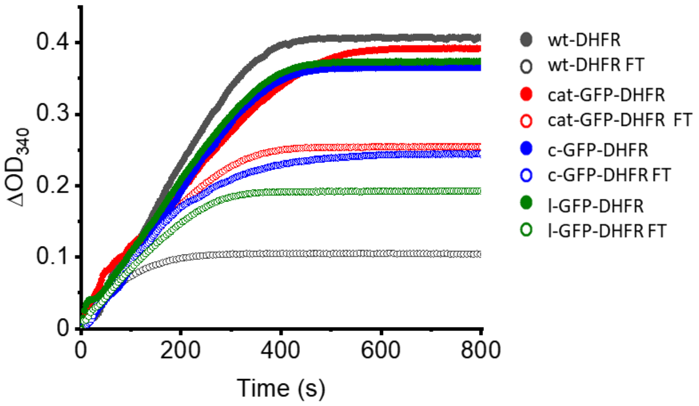

Supplementary Fig. 21 | DHFR catalytic activity assay after freeze-and-thaw-induced denaturation. The kinetic activity of wt-DHFR (black), cat-GFP-DHFR (red), c-GFP-DHFR (blue), and l-GFP-DHFR (green) before (solid circle) and after freeze-thawing (FT for short, hollow circle). The linear region of kinetic activity plots indicated the rate of the catalytic process.

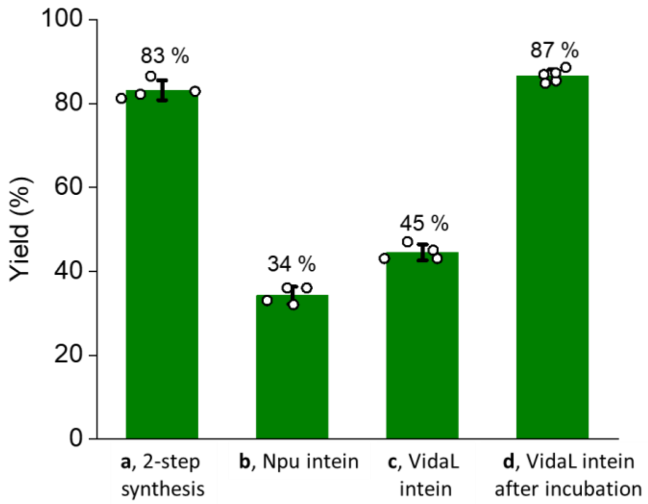

Supplementary Fig. 22 | Yields of cat-GFP synthesis using different strategies. a, The two-step synthesis using sortase cyclization. The yield is the sum of [2]cat-GFP and [3]cat-GFP after Ni-NTA purification. **b,** Direct synthesis of cat-GFP in cellulo using Npu intein. **c,** Direct synthesis of cat-GFP in cellulo using VidaL intein. **d,** cat-GFP using VidaL intein after high temperature incubation. The yields were defined as the ratio of catenane/(catenane+c-GFP1) and determined by SDS-PAGE and gel densitometry. The protein mass yields were approximately 18-20 mg/L. Experimental data points and error bars are presented as mean ± standard deviation, n = 4 independent experiments for **a**, **b**, and **c**, and n = 5 independent experiments for **d**.

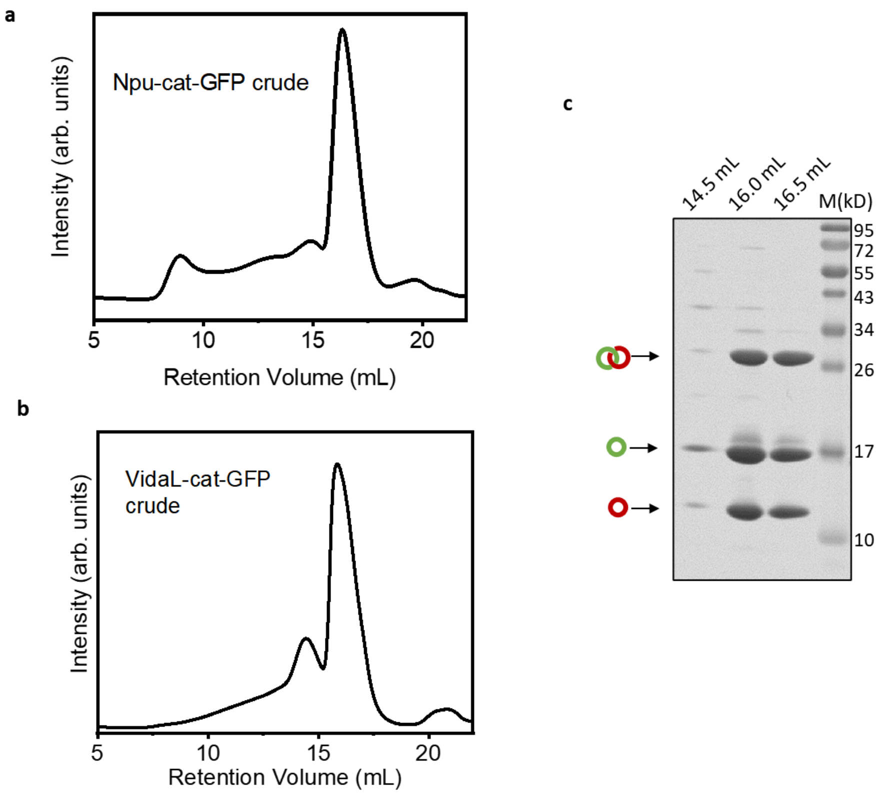

Supplementary Fig. 23 | Characterizations of cat-GFP purification. a, SEC trace of Npu-cat-GFP crude products after affinity purification. **b,** SEC trace of VidaL-cat-GFP crude products after affinity purification. **c,** SDS-PAGE analysis of VidaL-cat-GFP crude showing that the cat-GFP could hardly be purified from the unlinked rings in a simple way under native conditions. Source data for SDS-PAGE are provided in the Source Data for Supplementary Figures.

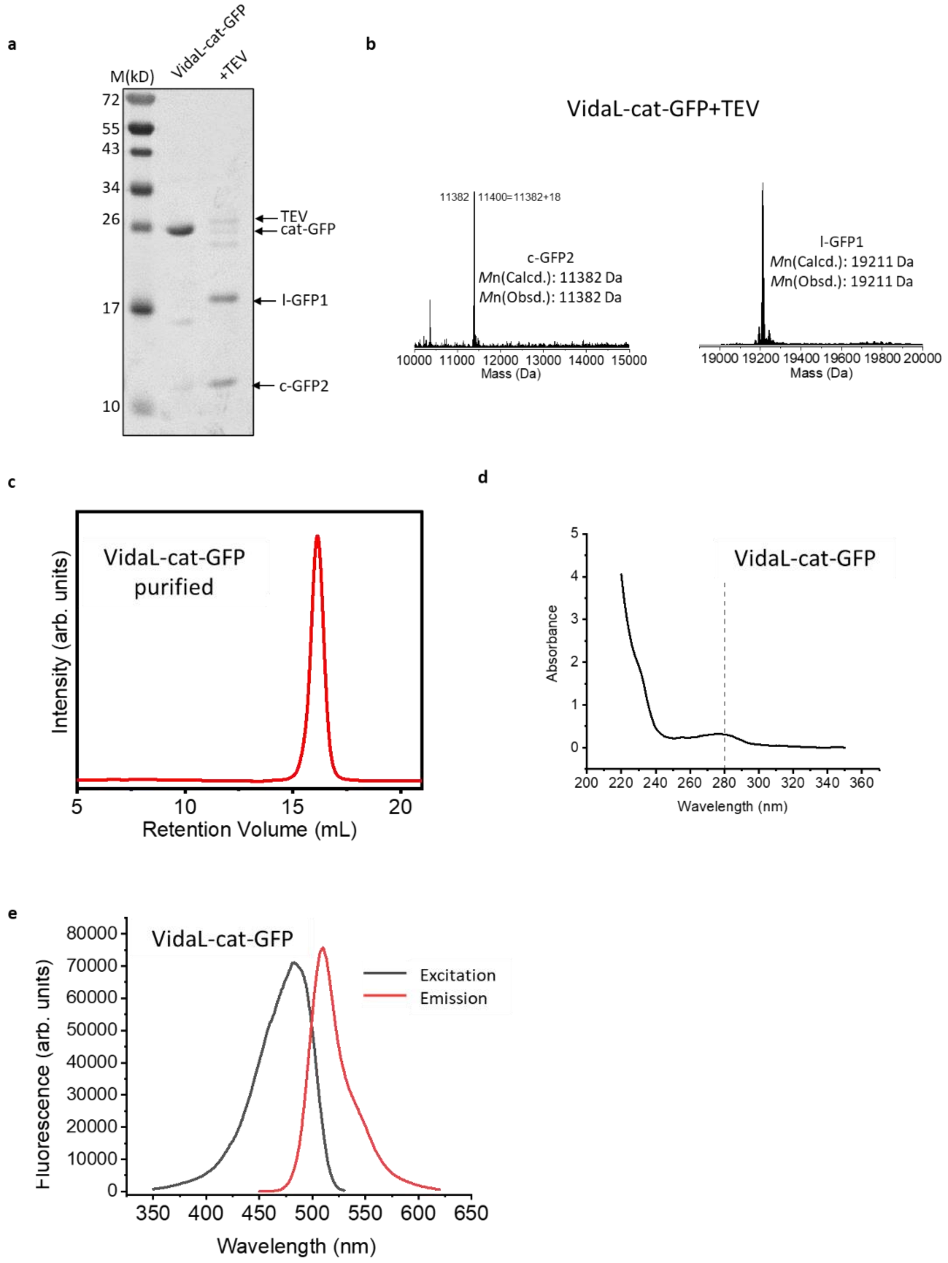

Supplementary Fig. 24 | Characterizations of VidaL-cat-GFP. a, SDS-PAGE analysis of the TEV protease digestion products of purified VidaL-cat-GFP. **b,** l-GFP1 and c-GFP2 produced by proteolytic digestion of VidaL-cat-GFP by TEV protease. **c,** SEC trace of the purified VidaL-cat-GFP after high-temperature incubation followed by dialysis. **d,** UV absorption spectrum of VidaL-cat-GFP. **e,** Emission spectrum (red) and excitation spectrum (black) of VidaL-cat-GFP. The VidaL-cat-GFP sample was adjusted to ~10 μM as determined by the absorbance at 280 nm, and then further diluted tenfold before fluorescence spectra measurement. Source data for SDS-PAGE are provided in the Source Data for Supplementary Figures.

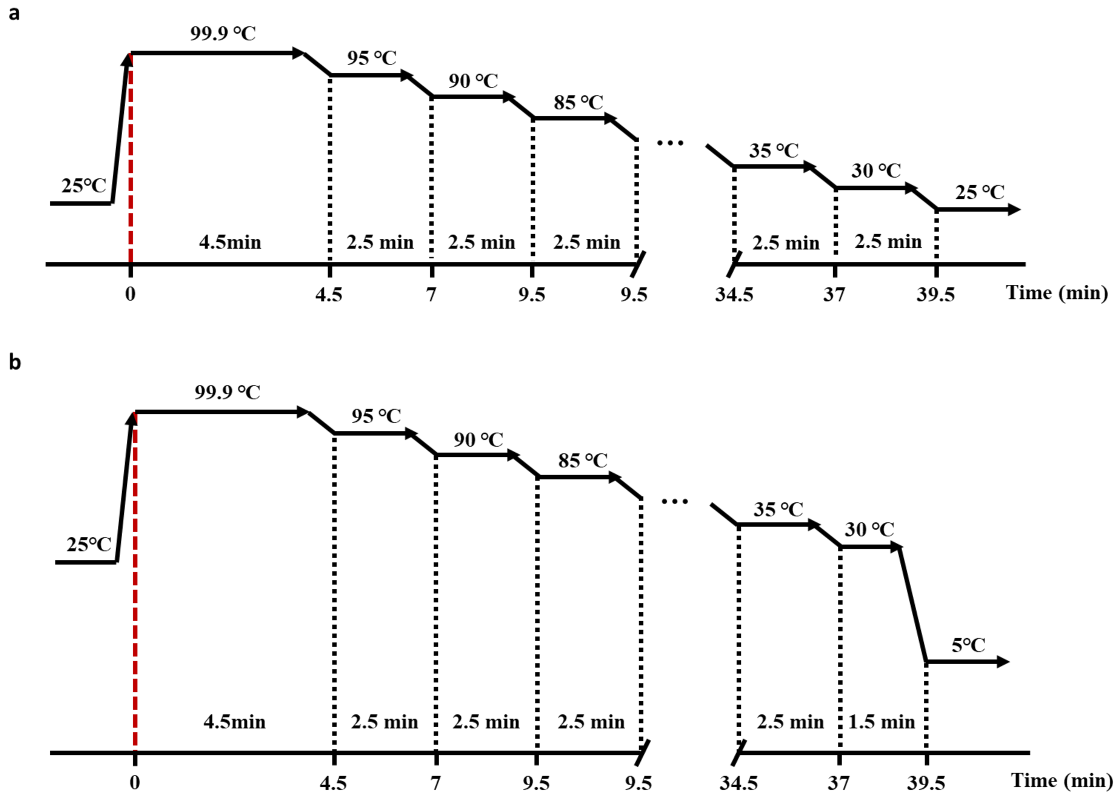

Supplementary Fig. 25 | Temperature settings in heat treatment and fluorescence recovery experiments. a, Temperature setting for slow recovery (cooled down to 25 ℃ used in cat-GFP, c-GFP, and l-GFP). **b,** Temperature setting for fast recovery (cooled down to 5 ℃ used in cat-GFP-POI, c-GFP-POI, and l-GFP-POI). The red line is the starting time point.

Supplementary Tables

Designs	Ring 1	Ring 2
A	1-2-3-4-5-6-7	8-9-10-11
B	1-5-6-7-8-9-10-11	2-3-4
C	1-2-6-7-8-9-10-11	3-4-5
D	1-2-3-4-5	6-7-8-9-10-11
E	1-7-8-9-10-11	2-3-4-5-6
F	1-2-4-5-6-7-8-9-10-11	3
G	1-2-3	4-5-6-7-8-9-10-11
H	1-11	2-4-5-6-7-8-9-10
I	1-2-3-4-5-6-11	7-8-9-10
J	1-2-3-4-11	5-6-7-8-9-10

Supplementary Table 1 | Single-domain GFP catenane designs. The number represents each GFP strand and "-" represents the connectivity of each strand that constituting each ring of catenane. Sequence information of Design A, B, and C are shown in Supplementary sequence 5 (only Design A, B, and C were preliminary synthesized), and topology diagram of each design are shown in Supplementary Fig. 2, respecitvely.

Proteins	$T_{m,\ GFP}$/°C	$T_{m,\ POI}$/°C
l-GFP	95.4	\
c-GFP	95.4	\
[2]cat-GFP	90.9	\
[3]cat-GFP	91.1	\
l-GFP-DHFR	95.5	50.7
c-GFP-DHFR	95.0	55.9
cat-GFP-DHFR	90.5	57.2
l-GFP-monellin	95.6	69.9
c-GFP-monellin	95.3	70.1
cat-GFP-monellin	90.4	73.1
l-GFP-leptin	95.7	55.0/41.9
c-GFP-leptin	95.2	51.6/42.8
cat-GFP-leptin	90.5	55.3/43.2
l-GFP-mCherry	96.9	
c-GFP-mCherry	96.7	
cat-GFP-mCherry	91.6	

Supplementary Table 2 | T_m values of single-domain GFP catenanes and the fusion derivatives. According to the official guide of MicroCal PEAQ-DSC, the measurement reproducibility gives an error of < 0.18 ℃ and the system reproducibility gives an error of < 0.1 ℃. Therefore, the total error of our experiment would be < 0.21 ℃ according to the error formula.

Note: DSC curves for samples containing leptin domain showed a broad bimodal peak. Since there are two cysteines that could form a disulfide bond in well-folded leptin, we infer that the two peaks represent leptin with or without the disulfide bond. According to Figure S3 in reference[5], the T_m value of wt-leptin is ~52 ℃.

Supplementary References

1. Wang, X.W. & Zhang, W.B. Cellular Synthesis of Protein Catenanes. *Angew. Chem. Int. Ed.* **55**, 3442-3446 (2016).
2. Liu, Y. et al. Cellular Synthesis and X-ray Crystal Structure of a Designed Protein Heterocatenane. *Angew. Chem. Int. Ed.* **59**, 2-8 (2020).
3. Wu, W.H. et al. Higher Order Protein Catenation Leads to an Artificial Antibody with Enhanced Affinity and In Vivo Stability. *J. Am. Chem. Soc.* **143**, 18029-18040 (2021).
4. Da, X.D. & Zhang, W.B. Active Template Synthesis of Protein Heterocatenanes. *Angew. Chem. Int. Ed.* **58**, 11097-11104 (2019).
5. Haglund, E. et al. Uncovering the molecular mechanisms behind disease-associated leptin variants. *J. Biol. Chem.* **293**, 12919-12933 (2018).

Source Data for Supplementary Figures

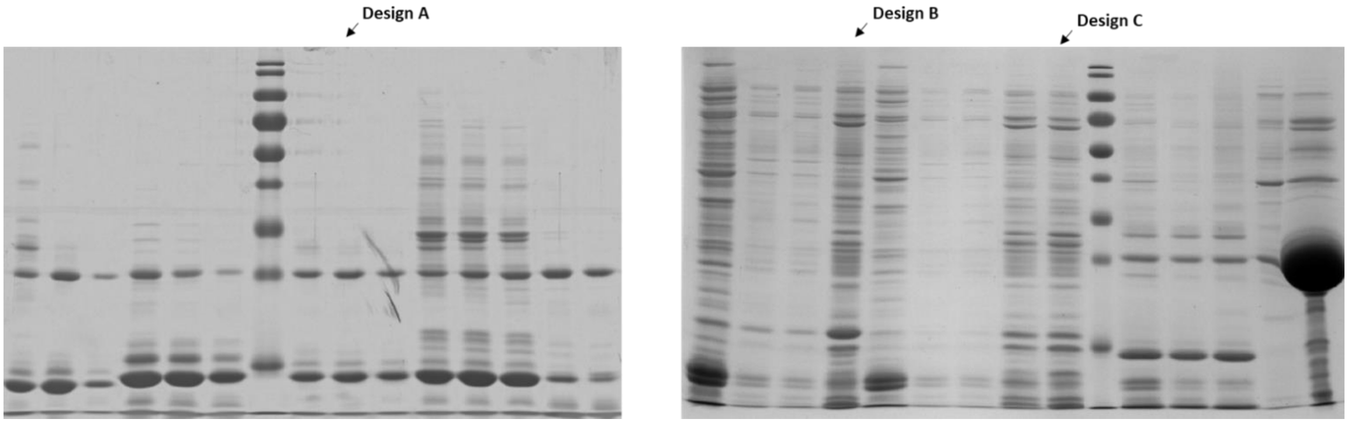

Source Data for Supplementary Fig. 2. Non-cropped image of SDS-PAGE of Supplementary Fig. 2k.

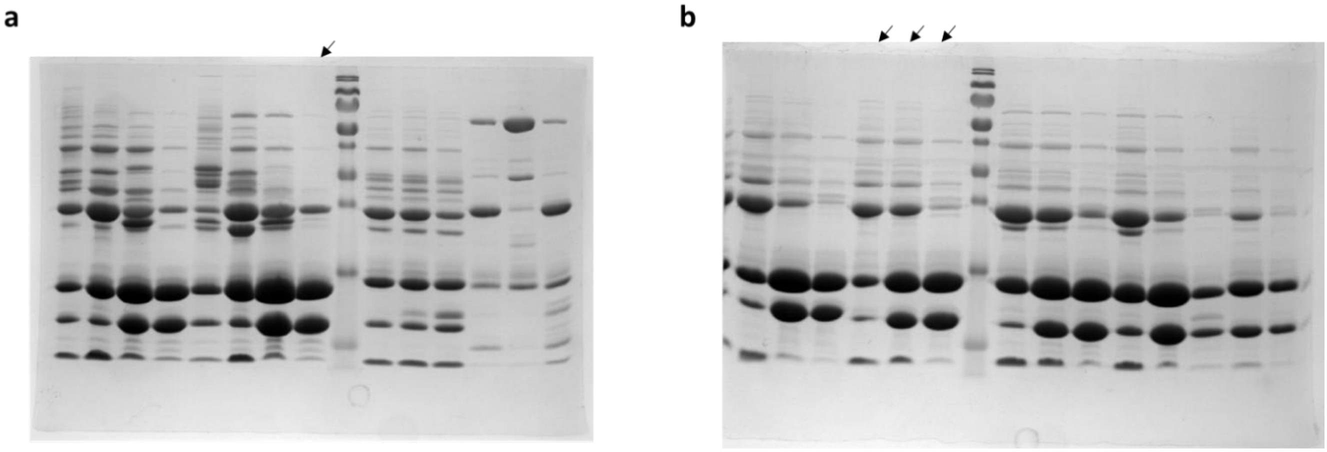

Source Data for Supplementary Fig. 3. **a,** Non-cropped image of SDS-PAGE of Supplementary Fig. 3a. **b,** Non-cropped image of SDS-PAGE of Supplementary Fig. 3c.

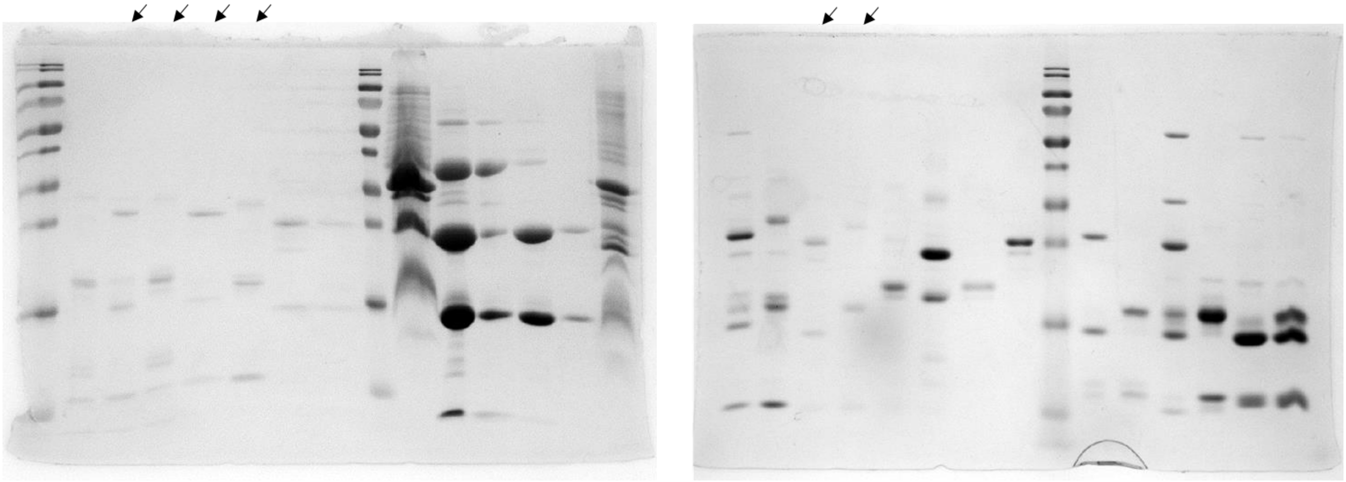

Source Data for Supplementary Fig. 7. Non-cropped image of SDS-PAGE of Supplementary Fig. 7a.

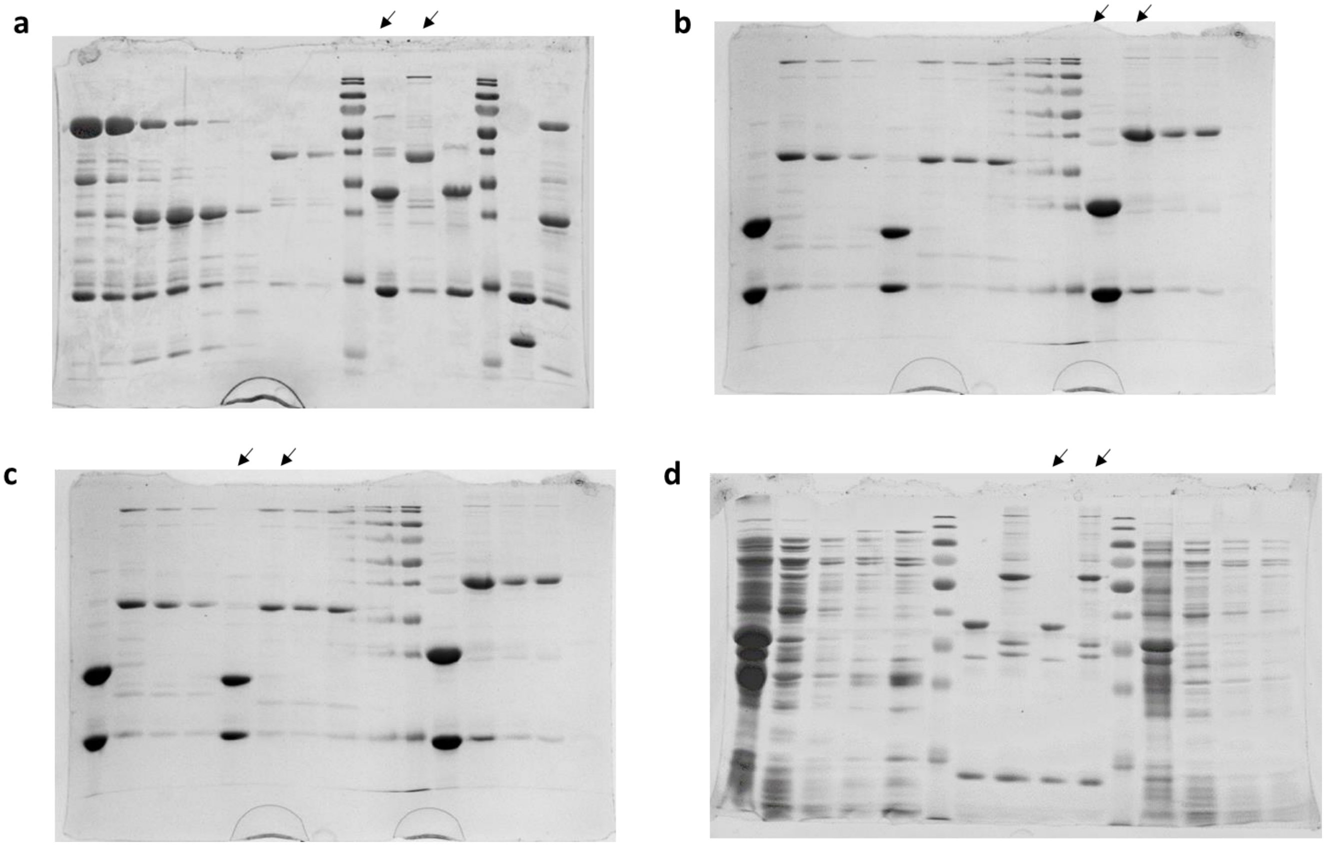

Source Data for Supplementary Fig. 9. **a,** Non-cropped image of SDS-PAGE of Supplementary Fig. 9b. **b,** Non-cropped image of SDS-PAGE of Supplementary Fig. 9e. **c,** Non-cropped image of SDS-PAGE of Supplementary Fig. 9h. **d,** Non-cropped image of SDS-PAGE of Supplementary Fig. 9k.

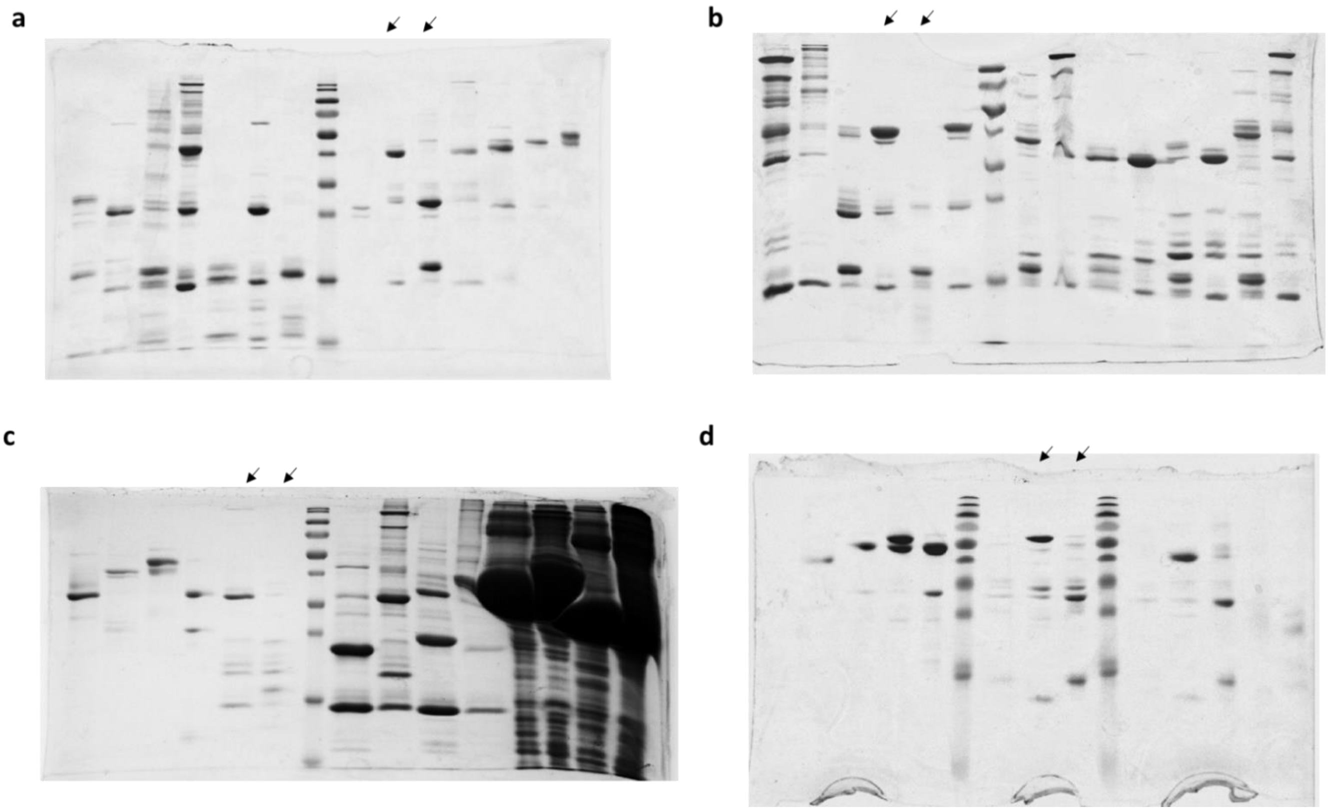

Source Data for Supplementary Fig. 10. **a,** Non-cropped image of SDS-PAGE of Supplementary Fig. 10a. **b,** Non-cropped image of SDS-PAGE of Supplementary Fig. 10c. **c,** Non-cropped image of SDS-PAGE of Supplementary Fig. 10e. **d,** Non-cropped image of SDS-PAGE of Supplementary Fig. 10g.

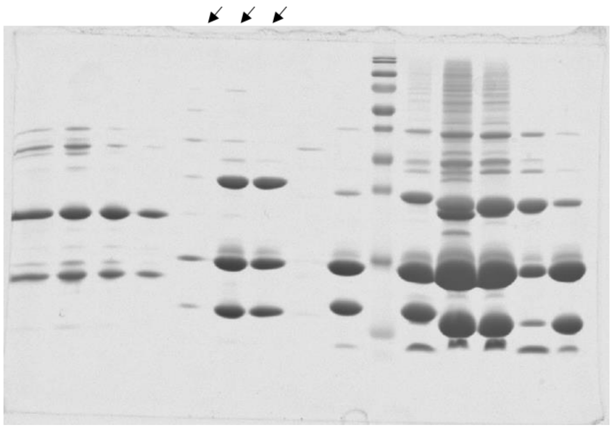

Source Data for Supplementary Fig. 23. Non-cropped image of SDS-PAGE of Supplementary Fig. 23c.

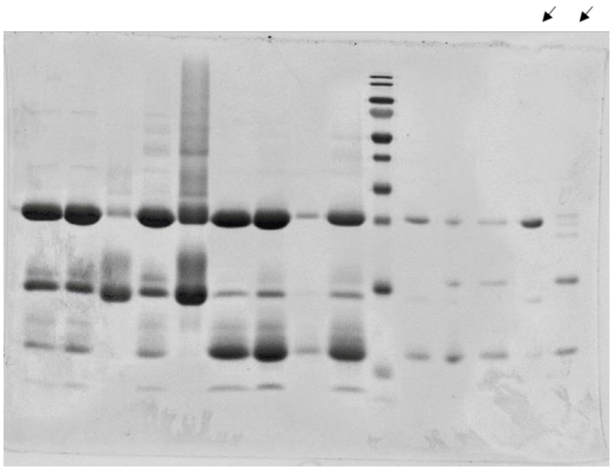

Source Data for Supplementary Fig. 24. Non-cropped image of SDS-PAGE of Supplementary Fig. 24a.
