# Supplementary material for: A single-domain green fluorescent protein catenane
Source: Nat Commun. 2023 Jun 13;14:3480. doi: 10.1038/s41467-023-39233-7 (PMC10264447; doi:10.1038/s41467-023-39233-7)
Supplement: Supplementary file 3 — Description of Additional Supplementary Files [file 41467_2023_39233_MOESM3_ESM.pdf]

Description of Additional Supplementary Files

File Name: Supplementary Data 1
Description: Sequences of oligonucleotides of primes used in this study
